# Unstructured network topology begets order-based representation by privileged neurons

**DOI:** 10.1007/s00422-020-00819-9

**Published:** 2020-02-27

**Authors:** Christoph Bauermeister, Hanna Keren, Jochen Braun

**Affiliations:** 1 Institute of Biology, Otto-von-Guericke University, Leipziger Str. 44, Haus 91, 39120 Magdeburg, Germany; 2 Center for Behavioral Brain Sciences, Leipziger Str. 44, 39120 Magdeburg, Germany; 3 Network Biology Research Laboratory, Electrical Engineering, Technion-Israel Institute of Technology, 3200003 Haifa, Israel

**Keywords:** Spiking networks, Neural code, Neural representation, Neural dynamics, Synchronization events, Leader neurons, Pioneer neurons, Motifs, Heterogeneous random connectivity

## Abstract

How spiking activity reverberates through neuronal networks, how evoked and spontaneous activity interacts and blends, and how the combined activities represent external stimulation are pivotal questions in neuroscience. We simulated minimal models of unstructured spiking networks in silico, asking whether and how gentle external stimulation might be subsequently reflected in spontaneous activity fluctuations. Consistent with earlier findings in silico and in vitro, we observe a privileged subpopulation of ‘pioneer neurons’ that, by their firing order, reliably encode previous external stimulation. We also confirm that pioneer neurons are ‘sensitive’ in that they are recruited by small fluctuations of population activity. We show that order-based representations rely on a ‘chain’ of pioneer neurons with different degrees of sensitivity and thus constitute an emergent property of collective dynamics. The forming of such representations is greatly favoured by a broadly heterogeneous connection topology—a broad ‘middle class’ in degree of connectedness. In conclusion, we offer a minimal model for the representational role of pioneer neurons, as observed experimentally in vitro. In addition, we show that broadly heterogeneous connectivity enhances the representational capacity of unstructured networks.

**Electronic supplementary material:**

The online version of this article (10.1007/s00422-020-00819-9) contains supplementary material, which is available to authorized users.

## Author summary

Simultaneous recordings of spiking activity from many neurons sometimes reveal subsets of privileged neurons that spike in a stereotypical order. Such repeating ‘motifs’ are expressed both by cortical networks in vivo and by cultured networks of cortical neurons in vitro, but neither their origin nor their function (if any) are understood. Here, we reproduce the dynamical features and representational capacity observed in vitro, with simulations of highly detailed, biologically plausible networks. Network connectivity was unstructured (random) and ‘broadly heterogeneous’, in that numbers of incoming and outgoing connections differed widely (and independently) between neurons. With this connectivity, numerous neurons were poised just below spiking threshold and retained full synaptic resources, thus being sensitive to one part, and influential on another part, of the network. During rising activity, these neurons funnel activity ‘many-to-one-to-many’, spiking in orderly sequences. Many different sequences can form, depending on the context of network activity at initiation. This explains both the emergence of repeating ‘motifs’ during periods of rising activity and the capacity of such ‘motifs’ to reliably and compactly represent gentle external inputs. We conclude that spiking ‘motifs’ in privileged neurons can emerge robustly and be highly informative, given sufficiently heterogeneous connectivity.

## Introduction

An important question in theoretical neuroscience is how externally evoked activity interacts with the spontaneous activity reverberating through neural networks. Closely related questions are how network topology shapes this interaction (touching on structure–function relationships) and how the blending of evoked and spontaneous activity represents external stimulation (touching on the ‘neural code’) (Rieke 2008; Decharms and Zador 2000; Thorpe et al. 2001; Ponulak and Kasinski 2011).

We address these issues by simulating in silico spiking neural networks (SNNs) with synaptic depression and different types of unstructured (random) connectivity. Although representing a drastic oversimplification of cortical networks in vivo, randomly connected SNNs have contributed considerably to our understanding of neural function (Shepherd 2003). They provide generic models for experimentally observed dynamics of cortical activity associated with phenomena such as short-term memory, attentional biasing, or decision-making (Rolls 2008; Rolls and Deco 2010). In addition, SNNs deepen our understanding of such phenomena because their activity dynamics can often be described analytically in terms of mean-field theory (Feng 2003; Gerstner et al. 2014).

Randomly connected SNNs have long been studied experimentally by harvesting, dissociating, and culturing mature cortical neurons in vitro on the substrate of multi-electrode arrays (MEA), so that a small fraction of spiking activity may be monitored ($O(0.1\%)$ of all neurons) (Morin et al. 2005; Marom and Shahaf 2002). The spontaneous activity of in vitro networks exhibits intrinsic fluctuations of all sizes, ranging from long quiescent spells to sudden synchronization events (‘network spikes’, NSs). Depending on the balance of excitation and inhibition, in vitro networks may operate near, below, or above a regime of self-organized criticality (SOC), where fluctuations are distributed in a scale-free manner (Bak et al. 1988; Jensen 1998; Beggs and Plenz 2003; Pasquale et al. 2008; Gigante et al. 2015). However, many experimental studies have elected to focus on a super-critical regime, in which periods of comparatively small activity fluctuations alternate with all-encompassing synchronization events.

Several recent studies have investigated the gradual build-up of activity immediately prior to synchronization events (NS), as this build-up exhibits interesting features with possible functional implications. Firstly, the growing activity propagates on reproducible paths, triggering a particular sequence of spikes in a certain subset of neurons (pioneer neurons) (Eytan and Marom 2006; Rolston et al. 2007). Secondly, the recruitment order of pioneer neurons is informative about prior perturbations by external stimulation (Shahaf et al. 2008). Specifically, when external stimulation is delivered to alternative sites, the stimulated site may be reliably decoded from the recruitment order of pioneer neurons (Kermany et al. 2010). Thirdly, the information encoded in the gradual build-up of activity may be propagated to other networks. For example, the stimulated site in an upstream in vitro network, which sparsely projects to a downstream in vitro network, may be reliably decoded from the activity of the latter network (Levy et al. 2012). Taken together, these observations raise the intriguing possibility that even unstructured neural networks express an order-based representation, encoding past external stimulation in the activity of a privileged class of pioneer neurons.

Repeating ‘motifs’ in the sequence of neuronal recruitment have been reported also in vivo in sensory cortex (Luczak et al. 2007; Luczak and Barthó 2012; Luczak and MacLean 2012), in prefrontal and parietal cortex (Peyrache et al. 2010; Rajan et al. 2016), and in hippocampus (Matsumoto et al. 2013; Stark et al. 2015). As the same ‘motifs’ appear in spontaneous and evoked activities, they are considered an emergent property of local circuits (Luczak and MacLean 2012; Rajan et al. 2016). The possible functional significance of reproducible spike ordering, for example in the formation of memory patterns, is an active topic of research (Contreras et al. 2013; Stark et al. 2015; Rajan et al. 2016).

Numerous theoretical studies have investigated the collective spiking dynamics of unstructured networks (Tsodyks et al. 2000; Brunel 2000; Loebel and Tsodyks 2002; Wiedemann and Lüthi 2003; Persi et al. 2004a, b; Vladimirski et al. 2008; Gritsun et al. 2008, 2010, 2011; Zbinden 2011; Masquelier and Deco 2013; Luccioli et al. 2014; Gigante et al. 2015). Typically, these studies have combined leaky integrate-and-fire neurons (Tuckwell 2005) with depleting ‘resources of excitability’, such as spike frequency adaptation (Koch 1999) or frequency-dependent synapses (Tsodyks et al. 1998). Different types of collective dynamics may be obtained (synchronous, asynchronous, critical, supra-critical, etc.), depending on balance of excitatory and inhibitory resources and the dynamics of resource depletion (Poil et al. 2012). Several studies have focused on the supra-critical regime characterized by large synchronization events (Tsodyks et al. 2000; Loebel and Tsodyks 2002; Wiedemann and Lüthi 2003; Vladimirski et al. 2008; Luccioli et al. 2014; Masquelier and Deco 2013; Gigante et al. 2015). Interestingly, even excitable systems that do not expressly incorporate depleting resources may produce ‘extreme’ activity events [(e.g. networks of FitzHugh–Nagumo units, Ansmann et al. (2013)].

The emergence of pioneer neurons was first predicted by Tsodyks et al. (2000). Ensuring heterogeneity by providing for different (effective) firing thresholds, these authors described a subpopulation of neurons that fires reliably during the build-up towards a synchronization event. Extending these results, pioneers with intermediate firing thresholds were shown to be critical for the generation of synchronization events (Vladimirski et al. 2008). More detailed investigations suggested that pioneers tend to combine low firing thresholds with unusually dense outgoing connectivity (Zbinden 2011). The formation of highly connected ‘leader’ neurons could be favoured by activity-dependent plasticity (Effenberger et al. 2015). In summary, previous work established pioneers as ‘a critical subpopulation of intermediate excitability that conveys synaptic drive from active to silent cells’ (Vladimirski et al. 2008).

The primary focus of the present work lies on the representational capacity of pioneer neurons and on the interaction between spontaneous activity dynamics and weak external stimulation. Specifically, we investigated the conditions under which unstructured networks express pioneer neurons that form an order-based representation of prior stimulation. Another difference to previous studies is that we compare the effect of different types of random connectivities on pioneer neurons and their representational capacity. In particular, we consider ‘homogeneous random’ (Erdös–Rényi) connectivity, ‘scale-free’ connectivity (Barabási and Albert 1999), and introduce a novel type of ‘heterogeneous random’ connectivity.

We show that the gradual build-up of activity towards a synchronization event proceeds in a reproducible manner, recruiting identifiable pioneer neurons in a particular order, consistent with experimental findings (Eytan and Marom 2006). We also show that this recruitment order is highly informative about the location of prior external stimulation, again consistent with experimental findings (Shahaf et al. 2008; Kermany et al. 2010). Additionally, we show that the formation of order-based representations is favoured by broadly heterogeneous connectivity, with a broad ‘middle class’ in terms of connectedness.

## Results

We begin by describing macroscopic dynamics of networks of spiking neurons and synapses with short-term plasticity, focusing on spontaneous fluctuations of activity and on the effect of gentle external stimulation (Sect. 2.1). Next, we characterize pioneer neurons in terms of their sensitivity to, and influence on, activity fluctuations and in terms of their contribution to network amplification (Sect. 2.2). We then compare the representation of gentle external stimulation by different aspects of population activity, including by pioneer neurons (Sect. 2.3). We then go on to show empirically that some types of random connectivity express the macroscopic and microscopic activity regimes in question more robustly and reliably than other types (Sect. 2.4). Finally, we investigate the reasons why broadly heterogeneous connection topologies favour order-based representations by pioneer neurons (Sect. 2.5).

### Macroscopic behaviour

We studied networks of leaky integrate-and-fire neurons with different kinds of random connectivities. Excitatory and inhibitory synapses were conductance-based with short-term depression and facilitation (Tsodyks et al. 1998). Networks were small and comprised 400 excitatory and 100 inhibitory neurons (see Sects. 4, 4.1, 4.1.1, 4.1.2).

This section describes spontaneous activity and activity evoked by gentle external stimulation. Following previous studies (Gigante et al. 2015; Tsodyks et al. 2000; Masquelier and Deco 2013; Loebel and Tsodyks 2002; Vladimirski et al. 2008; Wiedemann and Lüthi 2003; Luccioli et al. 2014), we focused on network architectures that combine low average activity with bimodal activity fluctuations (‘all-or-none’ synchronization events). Many interesting and instructive analyses can be anchored on the synchronization events expressed in this super-critical dynamical regime.

To understand qualitative differences between connection topologies, we consider three types of random connectivity: homogeneous random networks of Erdös–Rényi type, scale-free networks with explicit hubs, and a novel type of ‘broadly heterogeneous’ networks without explicit hubs (see Sects. 4, 4.1, 4.1.3). For all network types, mean connection density was $20\%$.

#### Spontaneous activity

**Fig. 1:**
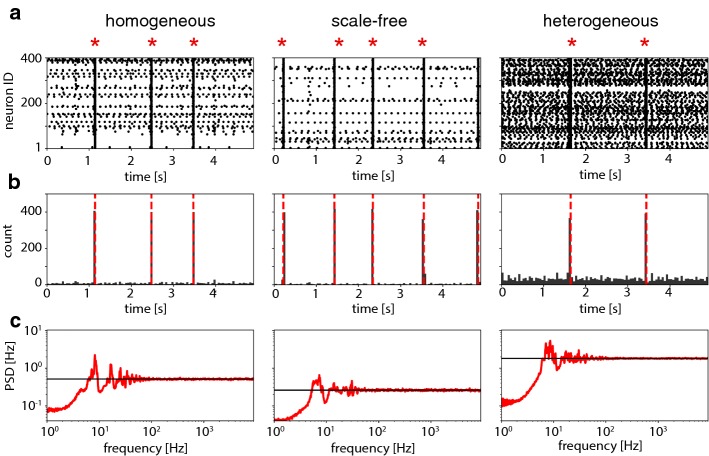
Spontaneous activity. **a** Spike rasters of excitatory neurons for the three network types (representative examples) with NS (red stars). **b** Corresponding spike counts (bin width $100\,\mathrm {ms}$) with NS (dashed red lines). **c** Power spectral densities of single neuron activity between NS, compared to Poisson spikes with the same average rate (black lines). The omission of NS periods is reflected in reduced power at low frequencies (colour figure online)

Representative periods of spontaneous activity are illustrated in Fig. 1a, b. The generally low level of activity is briefly interrupted by spontaneous synchronization events (network spikes, NSs), which recruit nearly all excitatory neurons at least once. Network spikes occur at somewhat irregular intervals (coefficient of variation $c_\mathrm {v}\approx 0.6$) and with frequencies $O(1\,\mathrm {Hz})$ (due to a suitable balance between excitation and inhibition, see Sect. 4). Although the network is deterministic, sparse connectivity ensures (apart from NS) irregular and asynchronous activity in many neurons (Brunel 2000; Mattia and Del Giudice 2002). The average power spectral density of individual neuron firing (apart from NS) is shown in Fig. 1c and resembles the power spectrum of Poisson spikes with a refractory period (which here corresponds to excised periods of NS) (Spiridon and Gerstner 1999). As random connectivity entails some non-uniformity in all three network types, the power spectral densities of individual neurons are quite diverse (Pena et al. 2018), with some neurons firing more frequently and regularly (albeit at different rates) and others firing more rarely and irregularly. Finite-size fluctuations of population activity (Brunel 2000; Mattia and Del Giudice 2002) induce pairwise correlations between individual neuron spikes (apart from NS), which on average are moderate for homogeneous and scale-free networks ($\rho _\mathrm {hom}=0.29\pm 0.21$, $\rho _\mathrm {sf}=0.18\pm 0.18$) and weak for heterogeneous networks ($\rho _\mathrm {het}=0.01\pm 0.013$).

**Fig. 2:**
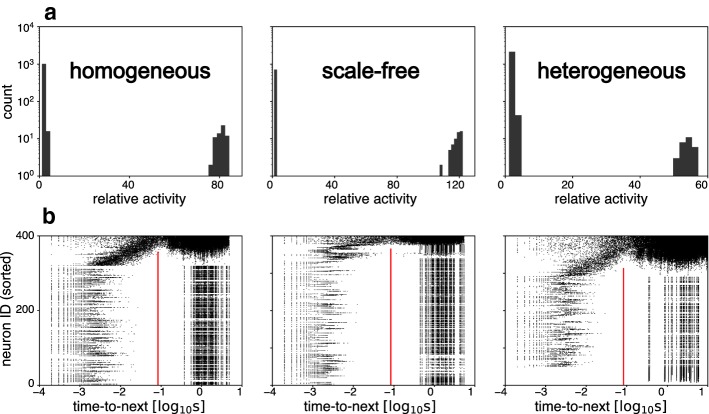
Bimodality of activity and relation of individual spikes to nearest NS. **a** Histogram of peak activation values during spontaneous activity fluctuations ($100\,\mathrm {s}$ simulations). Relative activity in multiples of mean firing rate $A_\mathrm {mean}$ (from left: $1.20\,\mathrm {Hz}$, $0.99\,\mathrm {Hz}$, and $1.43\,\mathrm {Hz}$, respectively). **b** Individual neuron spikes, relative to the next NS, during $100\,\mathrm {s}$ of spontaneous activity. Excitatory neurons are sorted vertically by mean firing rate (sorted neuron ID), with the least active neuron at bottom and the most active neuron on top. Individual neuron spikes are represented by black dots. For most neurons, spikes fall into two distinct classes: shortly before or long before the next NS (left and right columns of black dots, respectively). A heuristic latency criterion ($t_0=-80 \,\mathrm {ms}$, red lines) readily distinguishes these classes. Thus, the ‘first spike during a NS’ is well defined (i.e. rightmost dots in left column), for all but the most active neurons (colour figure online)

A telling characteristic of spontaneous population activity is the size distribution of positive fluctuations (Fig. 2a). Following earlier studies, we investigated networks with bimodally distributed fluctuations, where larger fluctuations constituted NS and distinctly smaller fluctuations represented the intervening periods. This bimodal dynamics allowed NS to be identified unambiguously. In heterogeneous random networks, larger and smaller fluctuations were separated less widely than in other network types. Rates of excitatory neuron spikes and NS were comparable ($2.1 \pm 3.0\,\mathrm {Hz}$ and $1.7 \pm 3.1\,\mathrm {Hz}$, respectively). In homogeneous random and scale-free networks, NSs were considerably larger, with many neurons contributing multiple spikes to each NS. Accordingly, the rate of neuron spikes ($1.0 \pm 1.5\,\mathrm {Hz}$ and $1.2 \pm 1.1\,\mathrm {Hz}$, respectively) was several times larger than the rate of NS ($0.4 \pm 1.6\,\mathrm {Hz}$ and $0.2 \pm 1.1\,\mathrm {Hz}$). In all three network types, inhibitory neurons fired continuously at $30 \pm 1\,\mathrm {Hz}$.

#### Evoked activity

**Fig. 3:**
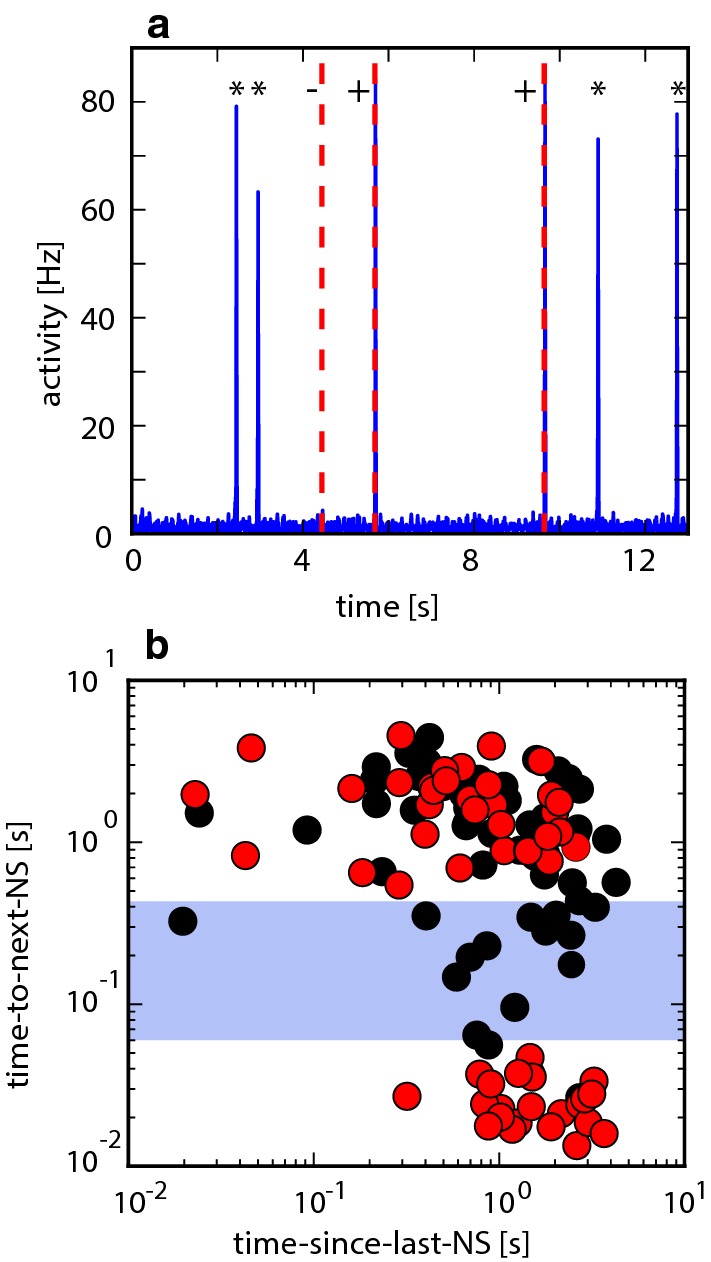
Evoked activity. **a** Superposition of spontaneous and evoked activities (example sequence). External stimulation forced simultaneous spikes in 5 randomly chosen excitatory neurons, at random time points (Poisson rate $0.4\,\mathrm {Hz}$), here marked by dashed red lines. Stimulation that succeeded (failed) to evoke a NS is marked by ‘$+$’ (‘−’). Spontaneous NSs are denoted by ‘$\star$’. **b** Classification of evoked and spontaneous NS. For each stimulation event, time to the next NS is plotted against time since last NS (red dots). For comparison, a null distribution is shown for an identical number of randomly timed, surrogate events (black dots). Stimulation events (red dots) form two distinct clusters (above and below blue bar), permitting us to classify stimulation events with high probability as either successful (below) or unsuccessful (above). In contrast, surrogate events are distributed continuously. Based on $120\,\mathrm {s}$ simulation of a heterogeneous random network, with stimulation rate equal to spontaneous NS rate (colour figure online)

A representative combination of spontaneous and evoked activities is shown in Fig. 3a. External stimulation was delivered by forcing *simultaneous *spikes in a small number of excitatory neurons (see Sects. 4, 4.5). As the effectiveness of stimulation varied considerably with the number and identity of target neurons, we simulated multiple ($O(10^1)$) network realizations to ensure representative results. To keep evoked and spontaneous NS as comparable as possible, we opted for a comparatively ‘gentle’ stimulation targeting a small set of neurons ($O(10^1)$).

To classify NS as ‘evoked’ or ‘spontaneous’, we compared the times of stimulation to the times of preceding and succeeding NS (Gigante et al. 2014). Helpfully, when intervals to the succeeding NS (‘time to the next NS’) were plotted against intervals from the preceding NS (‘time since last NS’), two distinct clusters emerged (Fig. 3b, red dots), which was not the case for surrogate events (black dots). NS classified as ‘evoked’ clustered on the lower right (below blue band, *long after* the previous NS and *shortly after* stimulation), whereas NS classified as ‘spontaneous’ clustered on top (above blue band, *long after* stimulation). Presumably, this clustering arose because even unsuccessful stimulation consumed some synaptic resources, delaying subsequent spontaneous NS.

### Pioneer neurons

We now turn to microscopic activity of individual neurons, especially pioneer neurons. Although all network types express pioneer neurons, heterogeneous networks produce them in far greater number (see Sect. 2.4). Accordingly, pioneer neurons are most easily characterized in heterogeneous networks. For this reason, all simulations in this and the following section are based on heterogeneous networks.

Due to random variations of connectivity, excitatory neurons fire with consistently different mean rates. The least active neurons never discharge, many neurons fire one spike per NS, and the most active neurons fire several spikes per NS. In most neurons, nearly all spikes are associated with NS and very few spikes occur during intervening periods. Due to this close association with NS, one may identify the ‘first spike’ of a particular neuron within a particular NS, at least for all but the most active neurons. This is illustrated in Fig. 2b, which shows a raster of individual neuron spikes, relative to peak activity of the next NS. Individual neuron spikes are sorted vertically by mean firing rate (sorted neuron ID). Note that the resulting spike raster is reminiscent of the letter ‘$\pi$’. The ‘left leg’ comprises spikes shortly before (and thus associated with) the next NS. The ‘right leg’ comprises spikes long before the next NS and thus presumably associated with the last NS. For all but the most active neurons, the two ‘legs’ are distinct, so that ‘first spikes’ can be identified without ambiguity (rightmost spikes in ‘left leg’).

**Fig. 4:**
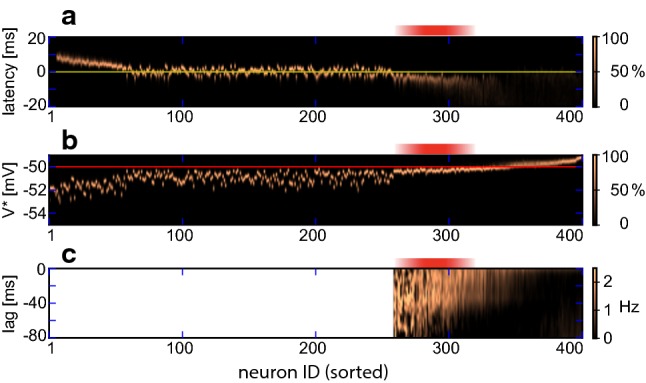
Latencies to NS, membrane voltage, and spike-triggered average activity. Excitatory neurons are sorted horizontally by mean activity (sorted neuron ID). Red shading marks the pioneer range (ID 260 to 320). **a** Latency of individual neuron first spikes, relative to the associated NS. Zero latency (yellow line) is defined by peak activity of the associated NS. Neurons in the pioneer range fire reliably before the NS (negative latencies). Colour scale indicates fraction of maximal density. **b** Distribution of $V^\star$ voltage in individual neurons, during intervals without NS, relative to firing threshold (horizontal red line). Neurons in the pioneer range have membrane potential just below threshold. Colour scale indicates fraction of maximal density. **c** Average deviation $\varGamma _\mathrm {i}(\tau )$ of population activity at lag $\tau$, conditioned on individual spikes of neuron *i* (see text and Sect. 4). Spikes of neurons in the pioneer range are consistently preceded by positive deviations. Note that deviation $\varGamma _\mathrm {i}(\tau )$ is not defined below ID 260 (colour figure online)

The first-spike latency of individual neurons, relative to the nearest NS, is illustrated in Fig. 4a. It is evident that firing latency decreases systematically with mean activity. The least active neurons consistently fire *after* NS (positive latencies, ID 6 to 55). Neurons with intermediate activity ($55<\mathrm {ID} < 260$) consistently fire *with* the NS (near-zero latencies). (The ordering of these neurons is effectively random, as they exhibit exactly identical levels of activity.) More active neurons ($260< \mathrm {ID} < 320$) fire consistently *before* the NS (negative latencies). The most active neurons ($320 < \mathrm {ID}$) fire at all times (both positive and negative latencies).

Accordingly, we tentatively identify ‘pioneers’ as neurons with a consistently negative spike latency relative to NS, in other words, with a standard deviation of latency smaller than the mean negative latency. Specifically, ‘consistency’ of latency can be defined as mean negative latency $\langle -\tau \rangle$ divided by standard deviation of latency $\mathrm {std}(\tau )$:

1 $$\begin{aligned} 1/\mathrm {CV}(\tau ) = \langle -\tau \rangle / \mathrm {std}(\tau ). \end{aligned}$$

In networks with heterogeneous connectivity, neurons with sorted IDs from approximately 260 to approximately 320 tend to have $1/\mathrm {CV}(\tau )\gtrapprox 1$ (Fig. 4a). This provisional criterion will suffice for heterogeneous networks. For the final two sections, where we will compare networks with different connectivities, we will redefine ‘pioneers’ in more general terms (see Sect. 2.4).

Why should firing latency grow more negative with higher mean activity? A simple explanation is differential ‘sensitivity’, that is, different probabilities that small fluctuations of population activity evoke a spike. More ‘sensitive’ neurons would be recruited more frequently and thus show higher mean activity. By the same token, more ‘sensitive’ neurons would be recruited earlier by the rising activity preceding a NS. Accordingly, the observed link between firing latency and mean activity is consistent with differential ‘sensitivity’.

To test this hypothesis, we established in simulations the individual distribution of membrane potential *V* for each excitatory neuron, during intervals without NS (absolute latency $|\tau | > 35\,\mathrm {ms}$). For convenience, we illustrate the results for a hypothetical potential $V^\star$, which is identical to *V* except in that it is never reset (and thus avoids discontinuities at threshold). As expected, the distribution of the $\star$ potential shifted systematically with mean activity (Fig. 4b). In the range of pioneers ($260< \mathrm {ID} < 320$), the mean value $\left\langle V^\star \right\rangle$ was just about one standard deviation below threshold voltage $V_\mathrm {th}$. Below this range, $\left\langle V^\star \right\rangle$ was consistently and well below threshold $V_{\vartheta }$, and above this range, $\left\langle V^\star \right\rangle$ is consistently and well above threshold.

The ‘sensitivity’ of neurons with $\left\langle V^\star \right\rangle < V_{\vartheta }$ may be quantified in terms of the standard deviation of star voltage, $\mathrm {std}(V^\star )$, relative to distance between its mean, $\left\langle V^\star \right\rangle$ and threshold $V_{\vartheta }$

2 $$\begin{aligned} \mathrm {CV}(V^\star ) = \mathrm {std}(V^\star )/[V_{\vartheta } - \left\langle V^\star \right\rangle ]. \qquad \qquad \end{aligned}$$

This measure will prove useful further below (see Sect. 2.5).

To further clarify the relation between individual neuron spikes and fluctuations of population activity, we computed the average population activity $\varGamma _i(\tau )$ preceding a single neuron spike, over and above mean population activity (see Sect. 4, Fig. 4c). To avoid contamination by NS, the analysis was based exclusively on periods *between* NS ($1000\,\mathrm {s}$ simulation). The results revealed that spikes of the earliest pioneers ($280<\mathrm {ID}<320$) were preceded by positive deviations of population activity (amplitude $\sim 1.5\,\mathrm {Hz}$, range $-60 \dots -40 \,\mathrm {ms}$), or in other words, by $\sim 30$ additional excitatory spikes over a $\sim 20\,\mathrm {ms}$ period.

**Fig. 5:**
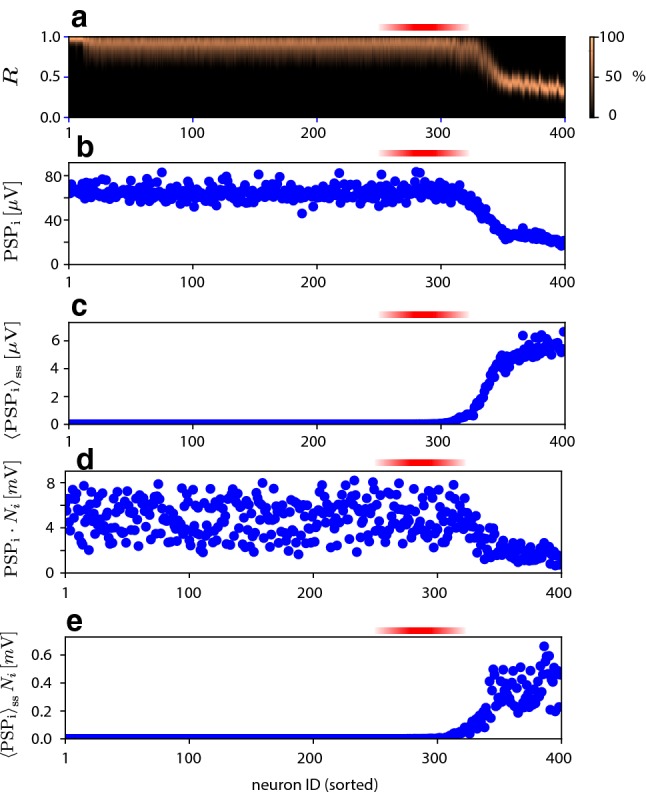
Influence of excitatory neurons. Red shading marks the pioneer range (ID 260 to 320). **a** Probability density of synaptic resources *R*, average over all efferent synapses of a given neuron, during intervals between NS. Resources decrease monotonically with mean activity. The most active neurons to retain substantial resources are neurons in the pioneer range. **b** Amplitude of post-synaptic potentials $\mathrm {PSP}_\mathrm {i}$ elicited by single spikes, averaged over all efferent synapses. **c** Steady-state post-synaptic potential $\left\langle \mathrm {PSP}_\mathrm {i}\right\rangle _\mathrm {ss}$ elicited by Poisson spiking at individual mean rate of neuron, averaged over all efferent synapses. Note that increasing firing rate overcompensates diminishing resources. **d** Same as (**b**), but *summed* over all efferent synapses. **e** Same as (**c**), but *summed* over all efferent synapses (colour figure online)

Pioneers may be not just sensitive to, but also influential on, population activity. To assess the potential influence of pioneers on population activity, we established the synaptic effectiveness over all efferent projections. As a first step, we computed the probability density of recovered synaptic resources for the efferent projections of each excitatory neuron, during intervals without NS (absolute latency $|\tau |>35\,\mathrm {ms}$, Fig. 5a). Unsurprisingly, synaptic resources proved to be more depleted in more active neurons. However, pioneers, which rarely fire between NS, retained at least 75 $\%$ of their synaptic resources. As a second step, we assessed the post-synaptic impact of a neuron by computing the average post-synaptic potential elicited by single spikes (Fig. 5b) and by repeated (Poisson) spikes (Fig. 5c) of the neuron in question (see Sect. 4, Spike-triggered population activity). Additionally, we also show (Fig. 5d, e) the *summed* effect on the post-synaptic potential of a single spike. The additional variability reflects the heterogeneity of connectivity (in- and out-degree). Neither analysis suggested that pioneer neurons are uniquely influential. However, the analysis did show pioneer neurons to be the most active and sensitive neurons that also retain substantial synaptic resources and therefore emit influential single spikes. In neurons that are even more active, the influence of single spikes is smaller, even though the combined influence of all spikes is larger.

Following (Zbinden 2011), we tried to relate the ‘influence’ of individual pioneer spikes on subsequent population activity and the number of efferent projections of the neuron in question. Although pioneers exhibited widely different influences, we found no straightforward relation to immediate connectivity (e.g. in terms of a standard definition of in-degree, out-degree, or ‘hubs’). In our hands, pioneer neurons exhibit intermediate degrees of both afferent and efferent connectivities (and certainly are not ‘hubs’ in the sense of (Wills and Meyer 2019)) (see also Fig. 12). Apparently, the ‘influence’ of pioneers depends on non-local connectivity spanning multiple sequential connections, both direct and indirect. This non-local connectivity is a network property and not straightforward to quantify.

**Fig. 6:**
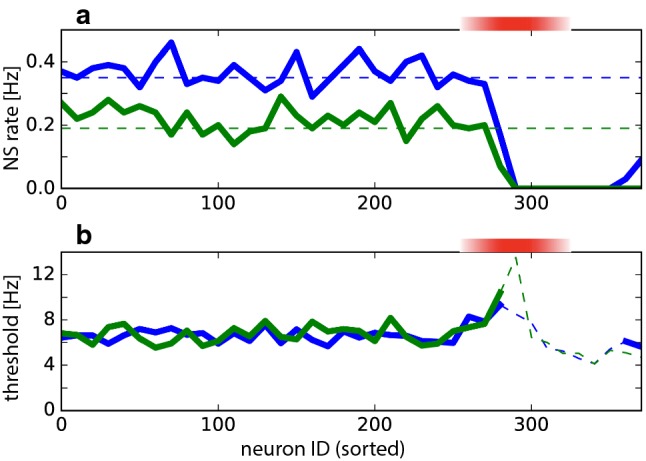
Effect of silencing groups of neurons. Red shading marks the pioneer range (ID 260 to 320). **a** Rate of NS as a function of *N*, for modified networks with neuron cohort $N \le \mathrm {ID} \le N+30$ silenced by de-efferentiation. Results are shown for two realizations (blue and green) with different spontaneous NS rates (dashed lines). NSs cease when neurons in the pioneer range are silenced. Typically, NSs are recovered when neurons above this range are silenced (e.g. blue trace). **b** Silencing pioneer neurons elevates threshold for triggering NS. Threshold population activity (in $\mathrm {Hz}$), after silencing neurons $N \le \mathrm {ID} \le N+30$. Two realizations are shown (blue and green traces). Over much of the pioneer range, only a lower bound for the threshold could be established (dashed traces), because even the largest observed fluctuations failed to trigger a NS (colour figure online)

In a further attempt to assess ‘influentialness’, we selectively silenced different groups of excitatory neurons (Tsodyks et al. 2000). Remarkably, silencing early pioneers ($290 \le \mathrm {ID} \le 320$) eliminated large synchronization events ($>14.5$ times mean activity), leaving only far smaller activity fluctuations ($<5.0$ times mean activity) (Fig. 6a). Silencing either less active neurons ($\mathrm {ID} < 290$) or more active neurons ($360 < \mathrm {ID}$) did not have this drastic effect. Evidently, pioneers were uniquely influential in terms of initiating large synchronization events. Interestingly, this cannot be attributed to disproportionate post-synaptic impact. As mentioned, pioneers were not exceptional when post-synaptic impact of Poisson firing was compared (Fig. 5c, d). Note, however, that such steady-state measures are unlikely to fully capture the ‘runaway’ dynamics of NS initiation.

To assess the possibility of differential contributions to the dynamics of NS initiation, we estimated thresholds for NS initiation in partially silenced networks (see Sect. 4). To this end, we established the bimodal distribution of peak activities during fluctuations of spontaneous activity (compare Fig. 2b), which reveals a low range of smaller fluctuations and a high range of full-blown NS. Threshold was defined as the largest observed value in the low range. Silencing a group of neurons reduces effective connectivity and recurrent amplification, which is expected to elevate threshold for NS initiation. If all neurons contribute similarly to amplification, silencing any group would elevate thresholds similarly. If some neurons contribute more than others, silencing some groups would elevate threshold differentially. The observed effect of silencing different groups of neurons is shown in Fig. 6b. Clearly, the threshold of NS initiation was elevated disproportionately by silencing pioneer neurons. Over much of the pioneer range, the threshold was elevated to ‘ceiling’, in the sense that even the largest observed activity fluctuations failed to trigger a NS.

We conclude that pioneers are exceptional in combining high ‘sensitivity’ to fluctuations of activity and in contributing prominently to network amplification. High ‘sensitivity’ is a consequence of the membrane potential hovering just below threshold, and large ‘influence’ is partially a consequence of largely recovered synaptic resources. However, the disproportionate contribution of pioneer neurons to the ‘runaway’ dynamics of NS initiation does not appear to be the result of any single neuron property (such as connectedness). This issue will be revisited in Sect. 2.5.

### Order-based representation

This section compares the representation of external stimulation by different aspects of neuronal activity associated with synchronization events (NS). Following (Kermany et al. 2010), we consider two spike-based and two rate-based encoding schemes by a set of *n* neurons (see Sects. 4, 4.5). The spike-based schemes are, firstly, the times $t_1, \ldots t_n$ of the first spike of each neuron following stimulation (‘spike times’) and, secondly, the rank order $o_1, \ldots o_n$ of the first spike of each neuron following stimulation (‘spike order’). The rate-based schemes are, thirdly, the mean spike rate $c_1, \ldots c_n$ of each neuron during the $100\,\mathrm {ms}$ following stimulation (‘neuronal rates’) and, fourthly, the temporal profile of the combined spike rate $r_1, \ldots , r_{50}$ of all *n* neurons during 50 successive time bins of $2\,\mathrm {ms}$ duration following stimulation (‘temporal rates’). For simplicity, we consider only stimulation attempts that were successful in eliciting a NS. The average activity following successful and unsuccessful stimulation is illustrated in Supplementary Figure.

External stimulation was delivered to $k=5$ groups of excitatory neurons, each comprising $s=10$ neurons chosen randomly. We refer to each group of target neurons as a ‘stimulation site’, although of course there is no spatial location in our simulated networks. Stimulation was delivered at random sites and random times reflecting a Poisson process (compare Fig. 3a). Stimulation rate was set at $1\,\mathrm {Hz}$, in order to obtain more evoked than spontaneous NS. Four heterogeneous networks were simulated over a duration of $300\,\mathrm {s}$. The classification of stimulation sites was non-trivial, because only synaptically mediated activity was analysed. The enforced spikes which constituted the stimulation were disregarded.

**Fig. 7:**
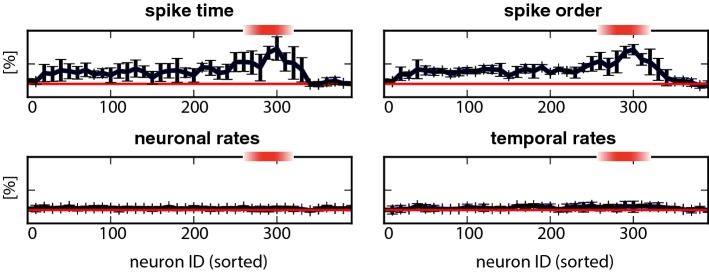
Classification performance of different decoding schemes, based on different groups of neurons. Red shading marks the pioneer range (ID 260 to 320). Results for ‘spike time’, ‘spike order’, ‘neuronal rates’, and ‘temporal rates’ are shown separately. Percentage of correct classification $\alpha (N)$ of one of the five stimulated locations is shown, based on the activity of neurons with sorted ID $[N,N+10]$. Chance performance is 20% (colour figure online)

To assess the quality of representation by different groups of neurons, we established classification performance separately for non-overlapping groups of $n=10$ activity-sorted neurons (sorted ID $[1,\ldots ,n]$, $[n+1,\ldots ,2n]$, $[2n+1,\ldots ,3n]$, and so on); see Fig. 7. Classification performance was far better for the two spike-based schemes (‘spike time’ and ‘spike order’) than for the two rate-based schemes (‘neuronal rates’ and ‘temporal rates’). Interestingly, classification performance peaked when decoding was based (in part) on pioneer neurons. Performance of the rate-based scheme barely exceeded chance.

**Fig. 8:**
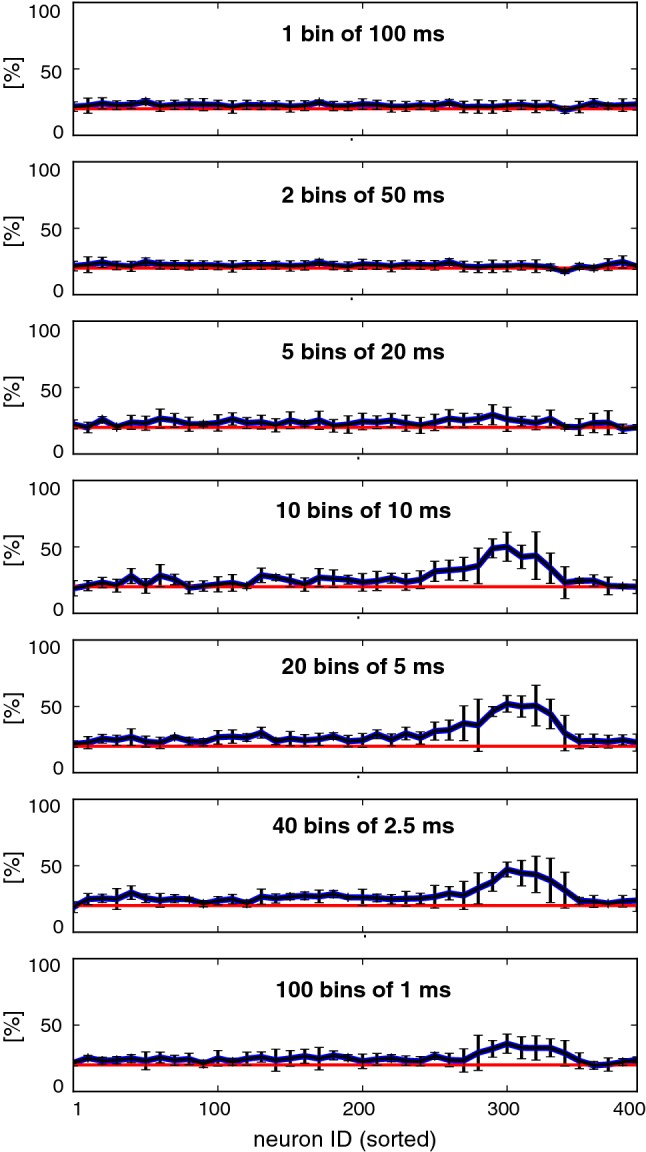
Interpolation between rate-based and spike-based decoding. The activity of groups of $n=10$ neurons over $100\,\mathrm {ms}$ was analysed in *k* time bins, forming a rate vector of length $k\cdot n$. Decoding performance is shown for different groups of neurons and different bin sizes

Interpolating between rate-based and spike-based decoding schemes, we divided the activity to be decoded ($n=10$ neurons over $100\,\mathrm {ms}$) into *k* time bins to obtain a rate vector of length *n* *k*. Performance in decoding stimulation site on the basis of this vector peaked at $k=20$ bins and for sets of neurons in the pioneer range (Fig. 8), demonstrating that decoding performance hinges on sufficient time resolution. In principle, performance is expected to stabilize for even higher resolutions (larger *k*). The diminishing performance for $k=40$ and $k=100$ is an artefact due to incomplete convergence of the classifier.

**Fig. 9:**
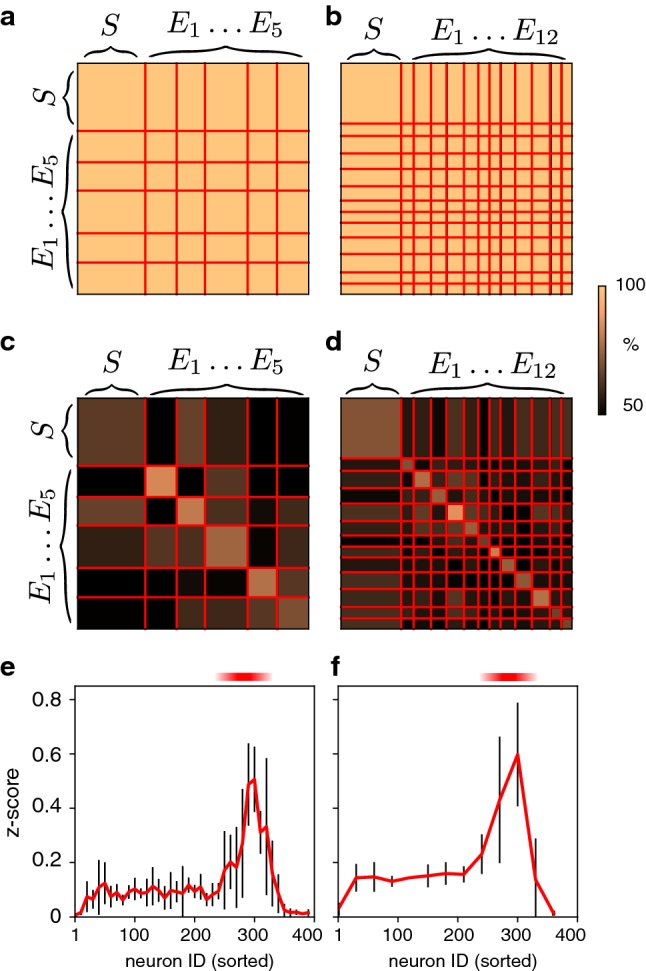
Matrices of average ‘spike order similarity’ (SOS) during different NS. Observed NSs were classified as ‘spontaneous’ (*S*), ‘evoked at site 1’ (${E}_1$), ‘evoked at site 2’ (${E}_2$), and so on, and class boundaries between sorted NS are marked by red lines. **a**–**d** Representative sets of non-pioneer or pioneer neurons, average SOS of pairs of NS (fraction of maximal similarity, colour scale) **a** Non-pioneer SOS, five stimulation sites ($k=5$ and $n=10$). **b** Non-pioneer SOS, twelve stimulation sites ($k=12$ and $n=30$). **c** Pioneer SOS, five stimulation sites ($k=5$ and $n=10$). **d** Pioneer SOS, twelve stimulation sites ($k=12$ and $n=30$). **e**, **f** Distance between SOS distributions (mean and standard deviation of *z*-score), within-class and between-class, for sets of neurons with contiguous ID starting with $N\in \{1, \ldots 393\}$. **e** Five stimulation sites and sets of ten neurons ($k=5$ and $n=10$, contiguous ID in range $[N,N+n-1]$). **f** Twelve stimulation sites and sets of thirty neurons ($k=5$ and $n=10$, contiguous ID in range $[N,N+n]$) (colour figure online)

The exceptional representational capacity of pioneer neurons became even more evident when the similarity of two ‘spike orders’ $o_1, \ldots o_n$ and $o'_1, \ldots o'_n$ was quantified. To this end, we modified the ‘Levenshtein edit distance’ (Levenshtein 1966) and measured ‘spike order similarity’ (SOS) by means of a measure based on permutations (see Sect. 4). Note that SOS may be computed for any pair of NS, spontaneous or evoked. Sorting all observed NS by class (‘spontaneous’, ‘evoked at site 1’, ‘evoked at site 2’, and so on), the results were collected into the average similarity matrices shown in Fig. 9a–d. To assess the representational capacity of pioneers, we computed such matrices from the SOS of both pioneers and non-pioneers. Non-pioneers were chosen randomly from the range [60, 260] and pioneers from the range [260, 320]). For non-pioneers, SOS was high for all pairs of NS, spontaneous or evoked (Fig. 9a, b), as these neurons spiked consistently and over a broad range of latencies (compare Fig. 4). In contrast, the SOS of pioneers was generally high for NS evoked at the same site and low for NS evoked at different sites (Fig. 9c, d). Clearly, pioneers were exceptional in that their ‘spike order’ was both unusually variable and unusually informative about stimulation site.

To corroborate these observations and to comprehensively compare all groups of excitatory neurons, we established the distribution of spike order similarity (SOS), both within and between classes of NS. As before, NS classes are understood to be ‘spontaneous’, ‘evoked at site 1’, ‘evoked at site 2’, and so on. Given two distributions of SOS values, with means $\mu _{1,2}$ and standard deviations $\sigma _{1,2}$, we expressed their ‘distance’ in terms of the *z*-score, $z ={|\mu _2 - \mu _1|}/({\sigma _1 + \sigma _2})$. The results are shown in Fig. 9e, f, for the two experiments with $k=5$ and $k=12$ stimulation sites, respectively. In both experiments, the difference between ‘within-class’ and ‘between-class’ distributions was most pronounced when SOS was computed for pioneers (ID 260 to 320). This demonstrates conclusively that the ‘spike order’ of pioneers was more informative about stimulation site than that of any other group of excitatory neurons.

The above results hold for networks with heterogeneous random connectivity. In analogous in silico experiments with homogeneous or scale-free networks, we encountered no significant representational capacity. Stimulation sites could not be decoded by either spike-based or rate-based encoding schemes, from either pioneer neurons or other groups of excitatory neurons. Although all network types expressed pioneer neurons, as defined by spike latency, only the pioneer neurons of heterogeneous networks appeared to form an order-based representation. To better understand the reasons why order-based representations are favoured by heterogeneous connectivity, we investigated and compared the macroscopic and microscopic dynamics of all three network types in more detail.

### Role of connection topology

To investigate the influence of connection topology on the macroscopic and microscopic activity described above, we simulated $\hbox {O}(10^4$) random networks with homogeneous, heterogeneous, and scale-free connectivity. Specifically, we systematically varied both absolute strength and relative strength of excitatory and inhibitory connectivity, choosing range of variation such that the desired macroscopic behaviour (all-or-none synchronization events) was covered in all three cases.

**Fig. 10:**
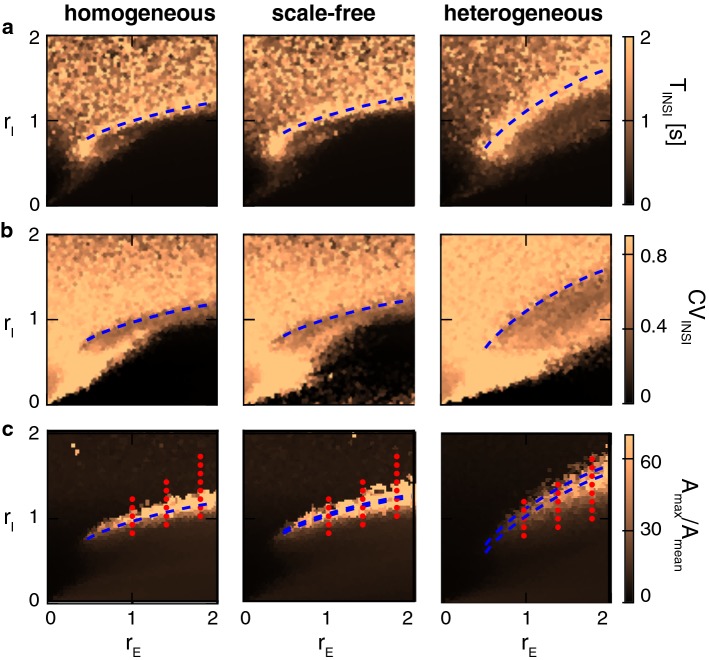
Macroscopic dynamics, excitation/inhibition strength, and type of connectivity. Dynamical characteristics of synchronization events, for different types of connectivity and for different absolute and relative strengths of excitation, $r_\mathrm{E}$, and inhibition, $r_\mathrm{I}$. Blue, dashed curves indicate the transitional region which, for each type of connectivity, separates the inhibition-dominated regime of ‘tonic’ dynamics from the excitation-dominated regime ‘asynchronous’ dynamics (see text). Red dots mark the $\left( r_\mathrm{E}, r_\mathrm{I}\right)$ value pairs further investigated in Fig. 11. **a** Average interval between NS, $T_\mathrm {INSI}$; **b** coefficient of variation of interval, $\mathrm {CV}_\mathrm {INSI}$; **c** activity ratio, $A_\mathrm {max}/A_\mathrm {mean}$ (colour figure online)

The combined effects of excitatory and inhibitory connection strength on macroscopic network activity are illustrated in Fig. 10. Strength of excitation and inhibition is expressed by multiplicative factors $r_\mathrm{E}$ and $r_\mathrm{I}$, respectively, relative to the connection strengths in a prototypical network. In other words, the four average connection strengths $\omega _\mathrm {ee}$, $\omega _\mathrm {ie}$ , $\omega _\mathrm {ei}$, and $\omega _\mathrm {ii}$, varied as $\omega _\mathrm {ee}=r_\mathrm{E}~{\bar{\omega }_{\mathrm {ee}}}$, $\omega _\mathrm {ie}=r_\mathrm{E}~{\bar{\omega }_{\mathrm {ie}}}$ , $\omega _\mathrm {ei}=r_\mathrm{I}~{\bar{\omega }_{\mathrm {ei}}}$, and $\omega _\mathrm {ii}=r_\mathrm{I}~{\bar{\omega }_{\mathrm {ii}}}$, where ${\bar{\omega }}_\mathrm {ee}$, $\bar{\omega }_\mathrm {ie}$ , ${\bar{\omega }}_\mathrm {ei}$, and $\bar{\omega }_\mathrm {ii}$ are the connectivities of a (suitably chosen) prototypical network. Note that this two-parameter variation of connectivity suffices to cover all relevant dynamical regimes (Gigante et al. 2015).

Macroscopic activity was characterized in terms of the average interval between consecutive NS, $T_\mathrm {INSI}$, the coefficient of variation of this interval, $\mathrm {CV}_\mathrm {INSI}$, and the ratio between maximal activity and mean activity, $A_\mathrm {max}/A_\mathrm {mean}$. The latter quantity is a convenient proxy for all-or-none synchronization events, as its value increases with the size and decreases with the frequency of such events. A value larger than thirty, $A_\mathrm {max}/A_\mathrm {mean} \gtrapprox 30$, was taken to indicate pronounced synchronization events (‘all-or-none’ NS). The values of $T_\mathrm {INSI}$ and $\mathrm {CV}_\mathrm {INSI}$ were obtained directly from the sequence of detected activity peaks (threshold $\theta = 0.5 A_{\mathrm {max}}$, see Sect. 4).

As shown in Fig. 10, all three types of connectivity exhibit a transitional regime (marked by blue dashed curves) for a certain balance of excitation and inhibition (‘E/I ratio’). In this transitional regime, synchronization events are infrequent (maximal $T_\mathrm {INSI}$) and occur at irregular intervals (large $\mathrm {CV}_\mathrm {INSI}$), and maximal activity far exceeds mean activity (large $A_\mathrm {max}{/}A_\mathrm {mean}$). These features resemble the experimentally observed activity of in vitro networks (Eytan and Marom 2006; Shahaf et al. 2008; Kermany et al. 2010).

Above the transition, dominant inhibition creates an ‘asynchronous’ regime, in which synchronization events are small, infrequent, and irregular (small $A_\mathrm {max}{/}A_\mathrm {mean}$, due to small $A_\mathrm {max}$). Below the transition, dominant excitation creates a ‘tonic’ regime, in which synchronization events are large, frequent, and regular (small $A_\mathrm {max}{/}A_\mathrm {mean}$ again, but now due to large $A_\mathrm {mean}$).

**Fig. 11:**
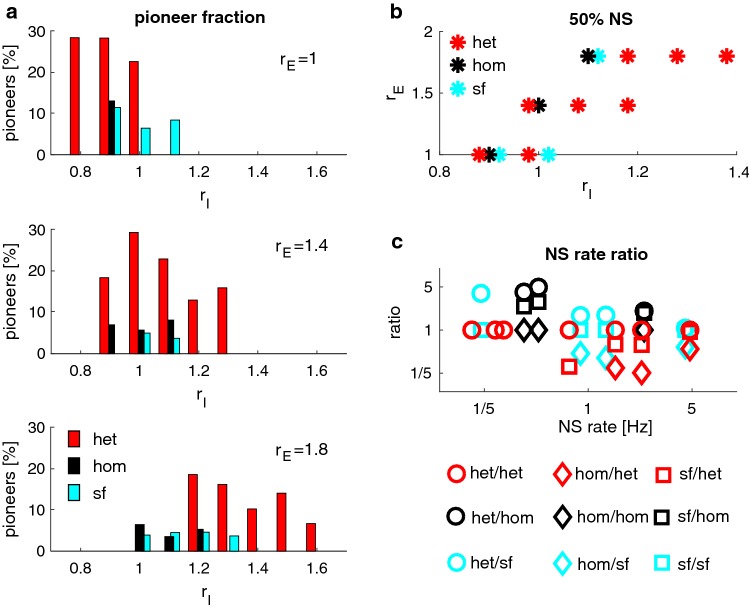
Macroscopic dynamics at selected connection strengths: $r_\mathrm{E}$ and $r_\mathrm{I}$. Fraction of pioneer neurons and NS rates in networks with $r_\mathrm{E} \in \{1.0, 1.4, 18\}$ and $r_\mathrm{I}\in \{ 0.8, 1.0, \ldots , 1.6, 1.8\}$ (red dots in Fig. 10c). **a** Percentage of pioneer neurons in networks of different topologies, for selected values $(r_\mathrm{E}, r_\mathrm{I})$ (see text). **b** Consistent NS in different network realizations. Value pairs $(r_\mathrm{E}, r_\mathrm{I})$ that produced NS in $\ge 50\%$ of realizations. **c** Disparity of NS rates in networks with different topologies, but identical values of $(r_\mathrm{E}, r_\mathrm{I})$. Ratio of NS rates, sorted by reference rate, for all ordered topology pairs. For example, at three identical value pairs $(r_\mathrm{E}, r_\mathrm{I})$, NS rates $f_\mathrm {hom}$ and $f_\mathrm {het}$ could be established for homogeneous and heterogeneous networks. Ratios $f_\mathrm {het}/f_\mathrm {hom}$ are shown against $f_\mathrm {hom}$ (black circles) and ratios $f_\mathrm {hom}/f_\mathrm {het}$ against $f_\mathrm {het}$ (red diamonds) (colour figure online)

To investigate the transitional region more closely, we simulated networks of all three types of connectivity at selected values, $\left( r_\mathrm{E}, r_\mathrm{I}\right)$, of relative connection strength (red dots in Fig. 10c). For each $\left( r_\mathrm{E}, r_\mathrm{I}\right)$ value pair and network type, we established the fraction of pioneer neurons (Fig. 11a). This fraction was consistently and significantly higher in heterogeneous networks than in homogeneous or scale-free networks. On average, this fraction was $22\%$ in heterogeneous networks, but only $11\%$ and $9\%$ in homogeneous and scale-free networks, respectively.

Here, we defined pioneer neurons in terms of a combined ‘consistency of latency’ criterion $\mathrm {CV}(\tau ) < 0.64$ and ‘sensitivity’ criterion $|\mathrm {CV}(V^{\star })| > 0.64$. This definition applies equally to all network types and agreed almost perfectly with our previous definition of pioneers (sorted ID from 260 to 320) in the case heterogeneous networks (see Fig. 13b). The additional ‘sensitivity’ criterion served to exclude neurons which fire ‘consistently’ around the peak of the NS.

Macroscopic dynamics differed not only between network types but even between different realizations of the same type, due to the randomness of connectivity. Figure 11b illustrates values $\left( r_\mathrm{E}, r_\mathrm{I}\right)$ for which a majority of realizations produced NS. It is evident that heterogeneous networks expressed NS over a broader range of $\left( r_\mathrm{E}, r_\mathrm{I}\right)$ values than homogeneous or scale-free networks.

Furthermore, different topologies expressed highly disparate dynamics for identical $(r_\mathrm{E}, r_\mathrm{I})$ values. For example, NS rates differed up to fivefold between topologies. Figure 11c illustrates the ratios of NS rates observed in all ordered topology pairs that expressed NS at identical $(r_\mathrm{E}, r_\mathrm{I})$ values.

In conclusion, all three types of connectivity expressed the dynamical regimes that are typical for excitatory–inhibitory networks (Gigante et al. 2015): (i) a transition region of balanced excitation/inhibition, with *large* synchronization events occurring infrequently and irregularly, (ii) an inhibition-dominated ‘asynchronous’ regime, with *small* synchronization events occurring infrequently and irregularly, and (iii) an excitation-dominated ‘tonic’ regime, *large* activity fluctuations arising frequently and regularly. However, there also were important quantitative differences. Firstly, heterogeneous networks featured a broader transitional region of ($r_\mathrm{E}, r_\mathrm{I}$) values, in which large synchronization events are expressed (Fig. 11b). Secondly, heterogeneous networks comprised a larger fraction of pioneer neurons ($\approx 20\%$) than homogeneous or scale-free networks ($\approx 10\%$) (Fig. 11a). Apparently, heterogeneous connectivity stabilizes both macroscopic and microscopic features of activity dynamics.

### Connection topology and order-based representation

The previous section investigated networks with identical connection strengths but rather dissimilar dynamics. As a complementary approach, we also investigated networks with similar macroscopic dynamics expressed by different connection strengths. Specifically, we chose excitatory and inhibitory connection strengths such as to ensure comparable rates of spontaneous activity ($\nu \approx 1\,\mathrm {Hz}/\mathrm {neuron}$) and network spikes (NSs) ($\nu _\mathrm {NS} \approx 0.6\,\mathrm {Hz}$) from all topologies (see Methods). Heterogeneous networks require significantly weaker excitation ($\omega _{{\mathrm {ee}},0}= 1.0\,\mathrm {nS}$) than homogeneous networks ($\omega _{{\mathrm {ee}},0}= 1.15\,\mathrm {nS}$) or scale-free networks ($\omega _{{\mathrm {ee}},0}= 1.45\,\mathrm {nS}$) to express similar macroscopic dynamics. Apparently, heterogeneous networks amplify activity fluctuations more effectively than other networks, so that smaller fluctuations suffice to trigger NS initiation. The reasons for this heightened sensitivity (amplification gain), and its relation to order-based representations, will become clear below.

Our analysis focused on the self-reinforcing build-up of activity immediately prior to synchronization events (NS), particularly on the orderly and reproducible recruitment of pioneer neurons during this build-up. Specifically, we examined the differences between network topologies (heterogeneous, homogeneous, scale-free) at three levels of description, proceeding from afferent connectivity to the distribution of membrane voltage and finally to the distribution spike times (relative to NS).

**Fig. 12:**
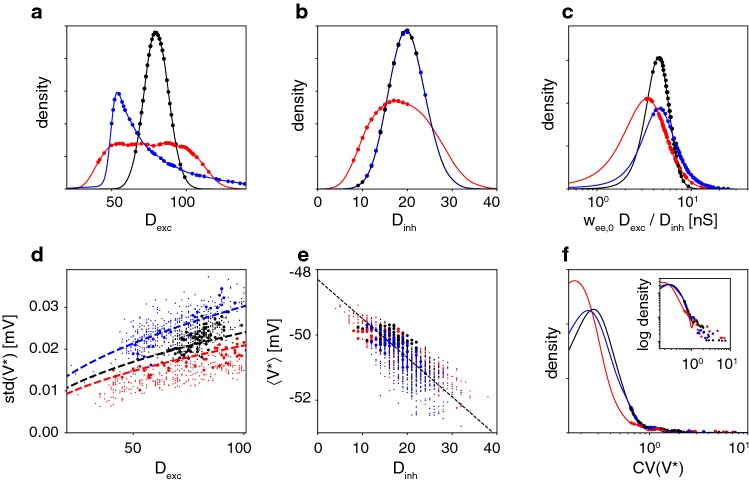
Afferent connectivity and star voltage $V^\star$ during periods without NS, for different network topologies. Colours distinguish heterogeneous (red), homogeneous (blue), and scale-free topology (black). Symbols mark pioneer neurons in a representative realization. **a** Distribution of afferent excitation, $D_\mathrm {exc}$, averaged over multiple network realizations. **b** Distribution of afferent inhibition, $D_\mathrm {inh}$, averaged over multiple network realizations. **c** Distribution of ‘effective afference ratio’, $\omega _\mathrm {ee,0} D_\mathrm {exc}/D_\mathrm {inh}$, averaged over multiple network realizations. **d** Dependence of the standard deviation of star voltage, $\mathrm {std}\left( V^\star \right)$ on $D_\mathrm {exc}$. Regression curves indicate proportionality $\mathrm {std}(V^\star ) \approx 2.1 \times 10^{7} \mathrm {\frac{V}{S}} \cdot \sqrt{D_\mathrm {exc}} \cdot \omega _{{\mathrm {ee}},0}$. **e** Dependence of mean star voltage, $\left\langle V^\star \right\rangle$, on $D_\mathrm {inh}$. Regression line indicates proportionality $\left\langle V^\star \right\rangle \approx -0.12\,\mathrm {mV} \cdot D_\mathrm {inh} - 48.3\,\mathrm {mV}$. **f** Distribution of sensitivity, $\mathrm {CV}(V^\star )$, with right tail on logarithmic scale (inset) (colour figure online)

We began by comparing the afferent connectivity of individual neurons and different network topologies. The number of excitatory and inhibitory inputs (‘excitatory in-degree’ $D_\mathrm {exc}$ and ‘inhibitory in-degree’ $D_\mathrm {inh}$) differed not only between neurons but also between topologies, as shown in Fig. 12a, b. Unsurprisingly, afferent connectivities were distributed more broadly in heterogeneous networks. The positions of neurons in the pioneer range (ranked ID 260 to 320) are marked for one representative heterogeneous network (red circles, Fig. 12a, b). As mentioned in Sect. 2.2, pioneers were *not* distinguished by exceptionally high degrees of connectivity and therefore were *not* ‘hubs’ in the sense of (Wills and Meyer 2019). If anything, pioneers were characterized by above average values of the ‘effective afference ratio’, $\omega _\mathrm {ee,0} D_\mathrm {exc}/D_\mathrm {inh}$ (Fig. 12c).

The afferent connectivity of a neuron determined the distribution of its membrane potential *V* or star voltage $V^\star$ (hypothetical membrane potential without spikes, established during periods without NS). Although the distribution of star voltage may depend on many factors, in our networks the standard deviation of $V^\star$ depended mainly on excitatory inputs (‘excitatory in-degree’ $D_\mathrm {exc}$), whereas the mean of $V^\star$ depended mainly on inhibitory inputs (‘inhibitory in-degree’ $D_\mathrm {inh}$), as illustrated in Fig. 12d, e. These approximate dependencies held for all network topologies (regression lines in Fig. 12d, e).

As discussed in Sect. 2.2, the probability that an increment of population activity triggers a particular neuron spike depended on the distribution $P(V^\star$), relative to firing threshold $V_{\vartheta }$, of the neuron in question. Neuronal ‘sensitivity’ may be quantified in terms of standard deviation of star voltage, $\mathrm {std}(V^\star )$, relative to its mean distance to threshold $V_{\vartheta }$ (Eq. 2). Crucially, neuronal sensitivity was distributed differently in networks of different topologies (Fig. 12f). In homogeneous and scale-free networks, the bulk of the neuronal population exhibited low sensitivity ($\mathrm {CV} \approx 0.0 - 0.4$) or intermediate sensitivity ($\mathrm {CV} \approx 0.4 - 1.0$), with only a small portion exhibiting high sensitivity ($\mathrm {CV} \gtrapprox 1.0$). In heterogeneous networks, an even larger fraction exhibited low sensitivity ($\mathrm {CV} \approx 0.0 - 0.4$), with a correspondingly smaller fraction of intermediate sensitivity ($\mathrm {CV} \approx 0.4 - 1.0$), but with a comparable number of neurons of high sensitivity ($\mathrm {CV} \gtrapprox 1.0$, inset in Fig. 12f). In all networks, the most sensitive neurons constituted the pioneers (symbols in Fig. 12f).

**Fig. 13:**
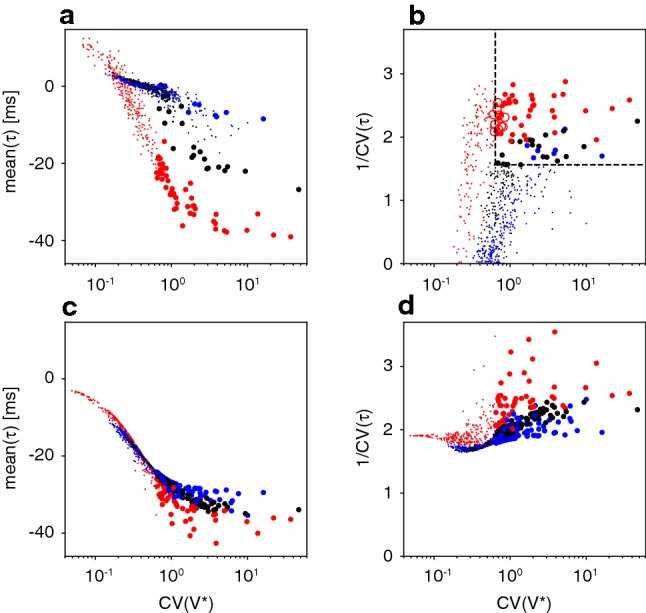
Spiking behaviour and sensitivity compared for different network topologies. Colours distinguish neurons of heterogeneous (red), scale-free (black), and homogeneous networks (blue). Pioneers are marked by large dots, other neurons by small dots. **a**, **b** Full effects of network topology (including different time courses of population activity). **a** Comparison of mean latency $\langle \tau \rangle$ during NS initiation and sensitivity $\mathrm {CV}(V^\star )$ during periods without NS. **b** Comparison of consistency of latency $1/\mathrm {CV}(\tau )$ and sensitivity $\mathrm {CV}(V^\star )$. The sensitivity consistency criterion (dashed lines) for pioneers and the sorted ID criterion largely overlap (discrepancies are marked by red circles). **c**, **d** Isolated effects of network topology (for identical time course of population activity). Corresponding comparisons of latency, consistency, and sensitivity when an identical, rigid time course of population activity is prescribed for all network topologies (colour figure online)

As illustrated in Fig. 13, spiking behaviour closely followed sensitivity $\mathrm {CV}(V^\star )$. On the one hand, the mean latencies increased systematically with sensitivity (Fig. 13a). Although this held for all networks, the relation was most pronounced in heterogeneous networks, where neurons with a broad range of sensitivities spiked over a correspondingly broad range of latencies. On the other hand, the consistency of latencies also increased systematically with sensitivity (Fig. 13b). This effect, too, was most pronounced in heterogeneous network. To summarize, we found that the most sensitive neurons fire both at the earliest latencies and with the highest degree of consistency.

The effect of the membrane potential distribution $P(V^\star )$ on the spike latency distribution $P(\tau )$ was most apparent in pioneer neurons of heterogeneous networks (red circles in Fig. 13a, b). Crucially, pioneers did *not* form a compact group clustered around particular values of sensitivity. Instead, pioneers were heterogeneous in that they were spread over a broad range of (high) sensitivity $\mathrm {CV}(V^\star )$, over a correspondingly broad range of negative spike latencies, $\left\langle -\tau \right\rangle$, but with a uniformly high consistency of latency, $1/\mathrm {CV}(\tau )$. In homogeneous and scale-free networks, the situation appeared different in that the bulk of the neuronal population clustered more narrowly with smaller mean latencies and lower consistency of latencies.

Thus, the distinguishing characteristic of heterogeneous networks appeared to be that neurons formed a continuous ‘chain’, which ranged from the highest sensitivity (and most negative, i.e. earliest, latency) to the lowest sensitivity (and latest latency). The neurons at the beginning of this ‘chain’ (highest sensitivity/earliest latency) also exhibited the highest consistency of latency (highest $1/\mathrm {CV}(\tau )$) and were the pioneers defined earlier (red circles in Fig. 13a, b). In other words, when a heterogeneous networks initiated NS, a ‘chain’ of pioneer neurons was recruited in a consistent rank order of decreasing latencies (from earliest to latest). In contrast, when other networks (homogeneous, scale-free) initiate NS, neurons were recruited less consistently and over a narrower range of latencies.

The above analysis showed a continuous ‘chain’ of neurons over all levels sensitivity in heterogeneous networks, but not in homogeneous or scale-free networks. The existence of this ‘chain of sensitivity’ explains both why pioneer neurons in heterogeneous networks are recruited in a reproducible rank order and why heterogeneous networks exhibited higher sensitivity (amplification gain) than other networks.

We conclude by highlighting a final emergent property of heterogeneous networks, namely the comparatively slow initiation of NS. As evident from Fig. 13a, the time course of NS initiation extended over approximately $40\,\mathrm {ms}$ in heterogeneous networks, compared to approximately $20\,\mathrm {ms}$ in homogeneous or scale-free networks. The comparatively slow initiation could have been due to a combination of factors, including the ‘chain of sensitivity’ and weaker excitation $\omega _\mathrm {ee,0}$. To verify that the ‘chain of sensitivity’ was a cause (and not a consequence) of slow initiation, we compared the microscopic dynamics of spike latencies in different network types for *identical* slow time courses of NS initiation. Specifically, we artificially prescribed a slow time course of population activity and predicted the resulting distributions of spike latencies, $p(\tau )$, with a simplified theory (see Sect. 4, toy theory for spike latencies). This revealed how connection topology ‘distorted’ the prescribed population time course.

The results are shown in Fig. 13c, d. The differential effects of connectivity, obtained with a rigid time course, agreed *qualitatively* with those observed for the native time course of each network type (Fig. 13a, b). In spite of the slow time course, neurons in homogeneous and scale-free networks expressed a narrower range of latencies (Fig. 13c), and a narrower range of consistency of latency (Fig. 13d), than neurons in heterogeneous networks. Thus, the characteristics of a continuous ‘chain’—from the highest to the lowest sensitivities and earliest to latest latencies, with the earliest latencies being also the most consistent—were less pronounced in homogeneous and scale-free networks, even when a slow time course was imposed. We conclude that the ‘chain of sensitivity’, which neurons formed in heterogeneous networks, is *not exclusively* a consequence of slower NS initiation. Instead, this ‘chain’ is presumably a contributing cause of slower NS initiation, together with weaker excitation. A more complete theory, which could more fully elucidate the consequences of network topology, remains a task for future work.

## Discussion

Over the past decade, compelling evidence has come to light that even unstructured networks of cortical neurons in vitro are capable of encoding and propagating information about past external stimulation (Eytan and Marom 2006; Shahaf et al. 2008; Kermany et al. 2010; Levy et al. 2012). Specifically, such networks appear to encode information in terms of the ordering of individual spikes from a privileged group of neurons, termed pioneer neurons. If even unstructured, in vitro networks—which, in contrast to structured cortical networks in vivo, have not been shaped by either neural development, sensory inputs, or reinforcement learning—possess such representational capabilities, this may have considerable implications for our general understanding of neural function. For cortical neuronal networks, in vivo might well subserve their individual functions roles by exploiting, extending, or customizing such intrinsic representational capabilities. Machine learning applications such as ‘reservoir computing’ further underline the functional possibilities of unstructured, recurrent networks, at least in combination with suitable decoding schemes (Maass et al. 2002; Jaeger and Haas 2004; Lukosevicius and Jaeger 2009).

Here, we show that the representational capabilities of unstructured networks observed in vitro emerge robustly under minimal assumptions. Firstly, we show that a network of excitatory and inhibitory spiking neurons with frequency-dependent synapses robustly expresses pioneer neurons, provided only that degree of connectivity varies broadly across the network. Secondly, we show that pioneer neurons reliably represent the site of prior external stimulation, in that the order of individual spikes depends characteristically on stimulation site. The same is not true for any other cohort of excitatory neurons. In a forthcoming publication, we will report additionally that sparse projections from ‘upstream’ to ‘downstream’ networks can efficiently propagate the information encoded by pioneer neurons (Bauermeister et al. 2015).

Several previous studies have thematized the emergence of pioneer neurons in unstructured networks (Tsodyks et al. 2000; Vladimirski et al. 2008; Zbinden 2011). In these studies, heterogeneity among excitatory neurons was obtained by means of variable (effective) firing thresholds, whereas connectivity remained homogeneously random (i.e. Erdös–Rényi type). Sorting excitatory neurons by average activity, Tsodyks and colleagues (Tsodyks et al. 2000) showed that a cohesive cohort of neurons with intermediate activity fires reliably in advance of large synchronization events (‘network spikes’, NSs). The same authors suspected these pioneer neurons to operate close to firing threshold and showed that the de-afferentiation of pioneer neurons reduces the rate of NS disproportionately. Vladimirski and colleagues (Vladimirski et al. 2008) analysed the effect of heterogeneous firing thresholds with a mean-field description of collective dynamics, confirming the importance of neurons with intermediate activity and demonstrating that heterogeneity ensures comparable dynamical instability in different network realizations. Finally, Zbinden (Zbinden 2011) sought to differentiate between neurons of intermediate activity in terms of afferent and efferent connectivity, reporting that influence on NS grows with the number of efferent projections.

Operationally, ‘pioneer neurons’ are defined as neurons that fire consistently during NS initiation (Fig. 4). Pioneer neurons are highly consequential for the macroscopic network dynamics (Tsodyks et al. 2000) and form a ‘a critical subpopulation of intermediate excitability that conveys synaptic drive from active to silent cells’ (Vladimirski et al. 2008). Our results confirm that pioneers enhance a network’s ability to amplify spontaneous fluctuations of population activity. When pioneers (but not other excitatory neurons) are silenced, the threshold for evoking NS is elevated dramatically, essentially eliminating NS from the spontaneous dynamics [(Tsodyks et al. 2000) and Fig. 6]. When connection topologies are compared that comprise pioneers in smaller or larger numbers, weaker excitatory coupling suffices for similar macroscopic dynamics when pioneers are numerous than when they are few (Sect. 2.5).

The distinguishing features of pioneer neurons are not readily apparent. Pioneers exhibit intermediate overall activity well as intermediate degrees of both afferent and efferent connectivities (Fig. 12). Thus, contrary to (Zbinden 2011), pioneers are not ‘hubs’ in any sense of the term (Wills and Meyer 2019).

The membrane potential of pioneers hovers just below firing threshold (Figs. 4b, 12e) with comparatively small variance (Fig. 12d). As a result, pioneers are sensitive to positive fluctuations of population activity (Fig. 4c). The degree of ‘sensitivity’ may be inferred from the time of spiking (negative latency) before a subsequent NS and grows with the coefficient of variation of the membrane potential (Fig. 13a). Importantly, pioneers spikes are not merely early but also highly consistent (smallest standard deviation of latency, see Fig. 13b).

The dynamical importance of pioneer neurons cannot be fully understood in terms of single neuron properties. To gain a fuller understanding, we compared pioneer neurons in the context of different connection topologies. Specifically, we considered two (opposite) extreme cases and one intermediate case of connectivity. In the intermediate case, different degrees of connectivity (from zero to maximum) were equally numerous in the network (‘heterogeneous random’ connectivity). In one extreme case, all neurons exhibited the same (average) degree of connectivity (‘homogeneous random’ or Erdös–Rényi connectivity). In the other extreme case, a small number of neurons exhibited exceptionally high connectivity, while the majority had below average connectivity (‘scale-free’ connectivity (Barabási and Albert 1999)).

All investigated connectivities generated network spikes (NSs) at certain ratios of excitation and inhibition, as well as a cohort of moderately active neurons firing consistently prior to NS (Figs. 10, 11). In quantitative terms, however, heterogeneous networks stood apart from the others: NSs were expressed at lower ratios of excitation to inhibition (Fig. 10c), indicating higher network sensitivity/amplification gain, and pioneer neurons were expressed numerously and consistently over a wider range of excitation and inhibition (Fig. 11a, b). In short, the macroscopic and microscopic phenomenology (i.e. NS and pioneers) was more reproducible over different levels of excitation/inhibition and over random realizations of connectivity.

Motivated by experimental evidence from unstructured networks in vitro (Kermany et al. 2010), we investigated the representational capacity of pioneer neurons. In heterogeneous random networks, the ordering of pioneer spikes during NS initiation was highly context-dependent: ordering was largely random before spontaneous NS, but turned more stereotypical before NS evoked by external stimulation (Fig. 9c, d). Moreover, stereotypical spike ordering was characteristic for each of several alternative stimulation sites, reliably identifying the stimulation site (Fig. 7). Other measures of population activity, such as the average activity profile of individual neurons (‘neuronal rates’) or the temporal profile of population activity (‘temporal rates’), were largely uninformative.

Surprisingly, spike ordering during NS initiation revealed a clear-cut difference between pioneer and non-pioneer neurons. Apart from pioneers, no other cohort of excitatory neurons showed any context dependence in their spike order. Instead, the recruitment order of other neuron cohorts remained highly stereotypical during NS initiation, whether ‘spontaneous’ or ‘evoked’ (Figs. 7, 9a, b).

In networks with homogeneous or scale-free connectivity, where pioneers are less numerous, no context dependence of pioneer recruitment could be observed. However, the comparison is awkward because different connectivities produce highly dissimilar macroscopic dynamics for given (average) connection strengths (Fig. 11c). To compare networks with similar macroscopic dynamics, we suitably adjusted excitatory connection strength, choosing less excitation for heterogeneous than for homogeneous and for scale-free connectivity (Figs. 12, 13).

This more careful comparison revealed that pioneers in heterogeneous networks did not constitute a uniform group, but rather formed a continuous ‘chain’ of neurons with different sensitivities (as measured by $\mathrm {CV}(V^\star )$, Fig. 13a). The beginning of this chain was formed by the neurons with highest sensitivity and earliest latency and its continuation by neurons with progressively lower sensitivity and later latency. Importantly, firing latencies were highly consistent along much of this chain (as measured by $1/\mathrm {CV}(\tau )$, Fig. 13b). The combination or early latency and high consistency explained why the ‘chain’ of pioneer neurons was recruited in a consistent rank order of decreasing latencies (from earliest to latest) during NS initiation (see below).

In homogeneous and scale-free networks, neuron properties were more uniform and the majority of neurons occupied a comparatively narrow range of both sensitivity and spike latency (Fig. 13a). Moreover, firing latencies were substantially less consistent (Fig. 13b). Taken together, this explained why pioneer neurons in other networks were *not* recruited in a consistent rank order.

Thus, representational capacity and context-dependent recruitment order are emergent properties of collective dynamics and a consequence of the ‘chain of sensitivity’ formed by pioneer neurons in heterogeneous networks. This ‘chain of sensitivity’ also explains why heterogeneous connectivity exhibits higher network sensitivity (amplification gain) than others, so that comparable macroscopic dynamics is obtained with weaker excitation.

**Fig. 14:**
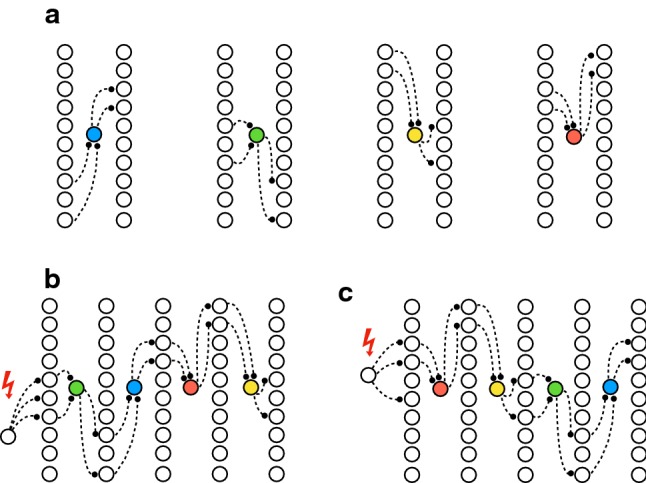
Many-to-one-to-many propagation of activity by pioneer neurons (highly schematic). **a** Four pioneer neurons are illustrated (blue, green, yellow, red), receiving afferent input from the left, and emitting efferent output to the right. Vertical columns of neurons represent the network as a whole. Afferent and efferent projections involve independent and random subpopulations of the network. **b** External stimulation (red bolt) of a specific subpopulation propagates, via a particular pioneer, to another subpopulation, starting an orderly sequence ($\hbox {green}\rightarrow \hbox {blue}\rightarrow \hbox {red}\rightarrow \hbox {yellow}$). **c** External stimulation of another subpopulation propagates via another pioneer, starting another orderly sequence ($\hbox {red}\rightarrow \hbox {yellow}\rightarrow \hbox {green}\rightarrow \hbox {blue}$) (colour figure online)

A schematic summary of our conclusion is illustrated in Fig. 14. The key point is that pioneer neurons propagate activity ‘many-to-one-to-many’. Many spikes are needed to elicit a single pioneer spike (e.g. approximately 30 additional spikes in the excitatory population precede each pioneer spike, Fig. 4c), which in turn elicits many subsequent spikes. As both afferent and efferent connectivities are partial and random, a pioneer may be sensitive to one subpopulation and may convey activity to an independent subpopulation (Fig. 14a). This ‘many-to-one-to-many’ propagation is effective only in a short window of time, before population activity has risen too high. During this window, pioneer spikes recruit subpopulations, which would in turn recruit other pioneer spikes, and so on, in a largely orderly sequence prescribed by afferent and efferent connectivity (Fig. 14b, c). In the absence of external stimulation, random fluctuations determine the initiation point of this orderly sequence, scrambling the recruitment order. In contrast, external stimulation prescribes a specific initiation point and produces a reproducible sequence which is informative about the stimulation site.

Note also that other excitatory neurons (non-pioneers) are less sensitive and recruited only when population activity is already higher and a NS is well underway (Fig. 4a, b). At this point, any information about NS initiation has been washed out by surging excitation. This explains why the majority of excitatory neurons spike with deterministic latencies, but carry little or no information about the site of external stimulation.

In conclusion, we have presented a minimal model for the representational capacity of a privileged class of neurons in unstructured networks, which provide a highly efficient order-based representation of external inputs. We term this model ‘minimal’ because it assumes only broadly heterogeneous connectivity in addition to standard neuron and synapse models (integrate-and-fire neurons and frequency-dependent conductance synapses). Thus, our results complement the results of other studies, which introduced heterogeneity by other means, for example inhomogeneous or dynamic background currents, or inhomogeneous or dynamic firing thresholds (Persi et al. 2004b; Gritsun et al. 2011; Masquelier and Deco 2013; Gigante et al. 2015; Rajan et al. 2016). Cortical networks in vivo presumably incorporate multiple kinds of heterogeneity, both in terms of connectivity (Landau et al. 2016) and in terms of resting potential or firing threshold (Harrison et al. 2015). Frequency-dependent synapses were necessary for obtaining for the supra-critical dynamics (i.e. large synchronization events) which is characteristic for in vitro networks (Eytan and Marom 2006; Shahaf et al. 2008; Kermany et al. 2010). Extending these results to the subcritical dynamics of in vivo networks presents an interesting challenge for future work (Priesemann et al. 2014).

We believe that our findings offer a deeper understanding of both the mechanisms underlying, and the possible functional significance of, repeating ‘motifs’ in the sequence of neuronal recruitment, as experimentally observed both in vitro (Eytan and Marom 2006; Rolston et al. 2007; Shahaf et al. 2008; Kermany et al. 2010) and in vivo in sensory cortex (Luczak et al. 2007; Luczak and Barthó 2012; Luczak and MacLean 2012), prefrontal and parietal cortex (Peyrache et al. 2010; Rajan et al. 2016), and in hippocampus (Matsumoto et al. 2013; Stark et al. 2015). We show how such repeating ‘motifs’ can result from a local interaction of cellular and synaptic conductances, as hypothesized by several authors (Luczak and MacLean 2012; Rajan et al. 2016), and demonstrate their potential functional significance as a highly compact and efficient representation of previous external inputs (Contreras et al. 2013; Stark et al. 2015; Rajan et al. 2016).

## Methods

### Network design and parameters

We simulated the collective activity of assemblies of 400 excitatory and 100 inhibitory neurons (leaky integrate-and-fire, LIF) (Tuckwell 2005), connected randomly by means of conductance synapses with short-term dynamics (Tsodyks et al. 1998). Spontaneous activity was evoked by a uniform and constant background current injected into all neurons. As neuron models were identical, the only source of heterogeneity was the connectivity ($20~\%$ mean density). Three types of connectivity were investigated: ‘homogeneous random’ (Erdös–Rényi), ‘scale-free’ (Barabási and Albert 1999), and ‘heterogeneous random’.

A neural simulator was programmed in C and verified against existing simulators, as well as by reproducing the results of (Tsodyks et al. 2000). Time was discretized in steps of $0.5\,\mathrm {ms}$, and numerical integration was performed with the first-order exponential integration method. To compute power spectra, smaller integration steps of $0.1\,\mathrm {ms}$ were used. To ensure representative results, we investigated multiple realizations of every network architecture (typically more than 10). Each type of connectivity expressed generally consistent behaviour, although event rates and average activity levels varied between realizations.

#### Neurons

The time-dependent membrane voltage *V* was governed by the differential equation

3 $$\begin{aligned} \frac{\mathrm {d} V}{\mathrm {d} t}(t)= \frac{E_\mathrm{L}-V(t)}{\tau _\mathrm{m}}+\frac{R_\mathrm{m} I_\mathrm{b}}{\tau _\mathrm{m}}+\frac{R_\mathrm{m} I_{\mathrm {syn}}(t)}{\tau _\mathrm{m}}, \end{aligned}$$

where $E_\mathrm{L}$ is the leak reversal potential, $\tau _\mathrm{m}$ is the membrane time constant, $R_\mathrm{m}$ is the membrane resistance, $I_\mathrm{b}$ is the background current, and $I_{\mathrm {syn}}$ is the synaptic current; see below. Whenever the voltage reached the threshold $V_{\vartheta }$, it was reset immediately to $V_{\mathrm {res}}$, where it remained for a refractory period $\tau _{\mathrm {ref}}$. The parameters of the neuron model were as follows: $E_\mathrm{L}=-70\,\mathrm {mV}$; $\tau _\mathrm{m} = 30\,\mathrm {ms}$ for excitatory neurons and $\tau _\mathrm{m} = 10\,\mathrm {ms}$ for inhibitory neurons; $R_\mathrm{m}=40\,\mathrm {M \Omega }$ for excitatory neurons and $R_\mathrm{m}=50\,\mathrm {M \Omega }$ for inhibitory neurons; $I_\mathrm{b}=525\,\mathrm {pA}$ for excitatory neurons and $I_\mathrm{b}=420\,\mathrm {pA}$ for inhibitory neurons; $V_{\vartheta }=-50\,\mathrm {mV}$; $V_{\mathrm {res}}=-65\,\mathrm {mV}$; $\tau _{\mathrm {ref}}=3\,\mathrm {ms}$ for excitatory neurons and $\tau _{\mathrm {ref}}=2\,\mathrm {ms}$ for inhibitory neurons. Note that the background currents raise the equilibrium potential over the threshold level, ensuring spontaneous activity (with hypothetical rates $\nu _\mathrm {exc}=12~ \mathrm {Hz}$ and $\nu _\mathrm {inh}=36~ \mathrm {Hz}$ in the absence of connectivity). Note further that the model is defined without noise. Initial membrane voltages were assigned randomly from the interval $[V_{\mathrm {res}},V_{\vartheta }]$. To avoid onset artefacts, the initial two seconds of activity were ignored.

#### Synapses

The synaptic state is described by four time-dependent variables (Tsodyks et al. 1998): the instantaneous fractions of recovered, active, and inactive (synaptic) resources (*R*(*t*), *E*(*t*), and *I*(*t*), respectively) and the fraction of resources *u*(*t*) recruited by presynaptic spikes. These non-dimensional variables satisfy the following equations:

4 $$\begin{aligned}&\frac{\mathrm {d} R}{\mathrm {d}t}(t) = \frac{I(t)}{\tau _\mathrm {rec}} - u(t+\epsilon )R(t-\epsilon ) \rho (t), \end{aligned}$$

5 $$\begin{aligned}&\frac{\mathrm {d} E}{\mathrm {d}t}(t) = - \frac{E(t)}{\tau _\mathrm{I}} + u(t+\epsilon )R(t-\epsilon ) \rho (t), \end{aligned}$$

6 $$\begin{aligned}&\frac{\mathrm {d} I}{\mathrm {d}t}(t) = \frac{E(t)}{\tau _\mathrm{I}} - \frac{I(t)}{\tau _\mathrm {rec}}, \end{aligned}$$

7 $$\begin{aligned}&R(t)+E(t)+I(t) = 1, \end{aligned}$$

8 $$\begin{aligned}&\frac{\mathrm {d} u}{\mathrm {d}t}(t) = \frac{-u(t)}{\tau _{\mathrm {facil}}} +U(1-u(t-\epsilon )) \rho (t), \end{aligned}$$

where $\rho (t):= \sum \nolimits _{i} \delta (t-t_i)$ is the Dirac comb associated with the spike train of the presynaptic neuron. The axonal conduction delay was uniform and $0.5\,\mathrm {ms}$. $\tau _\mathrm {rec}$ is the recovery time constant; $\tau _\mathrm{I}$ is the inactivation time constant; $\tau _{\mathrm {facil}}$ is the facilitation time constant; and *U* is a parameter associated with resource utilization. Parameter values are as follows (subscript ‘ee’ stands for ‘excitatory-to-excitatory’, ‘ie’ stands for ‘excitatory-to-inhibitory’, ‘ei’ stands for ‘inhibitory-to-excitatory’, ‘ii’ stands for ‘inhibitory-to-inhibitory’): $\tau _{\mathrm {I,ee}}=\tau _{\mathrm {I,ie}}=3\,\mathrm {ms}$ and $\tau _{\mathrm {I,ei}}=\tau _{\mathrm {I,ii}}=10\,\mathrm {ms}$; the values for *U*, $\tau _\mathrm {rec}$, and $\tau _{\mathrm {facil}}$ were randomly chosen and hence varied from synapse to synapse. Values were chosen from Gaussian distributions with mean $U_\mathrm {ee}=U_\mathrm {ei}=0.3$; $U_\mathrm {ie}=U_\mathrm {ii}=0.04$; $\tau _\mathrm {rec,ee}=\tau _\mathrm {rec,ei}=0.8\,\mathrm {s}$; $\tau _{\mathrm {rec,ie}}=\tau _{\mathrm {rec,ii}}=0.1\,\mathrm {s}$; $\tau _{\mathrm {facil,ie}}=\tau _{\mathrm {facil,ii}}=1\,\mathrm {s}$. The standard deviation of each distribution was half the respective mean. However, Gaussian distributions were clipped and restricted to a physically possible range ( i.e. positive values for time constants and values between zero and unity for *U*). For $\mathrm {ee}$- and $\mathrm {ei}$ synapses, $\tau _{\mathrm {facil}}$ was zero (no facilitation).

The synaptic current $I_{{\mathrm {syn}},i}$ of the *i*th neurons was

9 $$\begin{aligned} I_{{\mathrm {syn}},i}(t)=g_{{\mathrm {exc}},i}(t)(E_{\mathrm {exc}}-V(t))+g_{{\mathrm {inh}},i}(t)(E_{\mathrm {inh}}-V(t)), \end{aligned}$$

where the reversal potentials were chosen as $E_{\mathrm {exc}}=0$ and $E_{\mathrm {inh}}=-70\,\mathrm {mV}$. The conductances $g_{{\mathrm {exc}},i}$ and $g_{{\mathrm {inh}},i}$ are given by

10 $$\begin{aligned} g_{{\mathrm {exc}},i}(t)= & {} \sum \limits _{j\,\mathrm {exc}} w_{ij} E_{ij}(t) \end{aligned}$$

11 $$\begin{aligned} g_{{\mathrm {inh}},i}(t)= & {} \sum \limits _{j\,\mathrm {inh}} w_{ ij } E_{ ij }(t), \end{aligned}$$

where the sum is over all excitatory (inhibitory respectively) neurons. $w_{ ij }$ is the matrix of synaptic weights, and $E_{ ij }$ is the (time-dependent) matrix of resources in the active state.

The assignment of neuron and synapse parameters was modelled on (Tsodyks et al. 2000).

#### Connectivity matrix

In homogeneous random (Erdös–Rényi) networks, each ordered neuron pair (*i*, *j*) formed a synaptic connection $i\rightarrow j$ with $20~\%$ probability. Over all neurons, the degree of connectivity thus followed a Gaussian distribution. Scale-free networks were obtained with the ‘preferential attachment’ procedure (Barabási and Albert 1999), such that connectivity followed a power-law distribution with a mean connectivity of $20~\%$. Heterogeneous random networks were generated as follows. Every neuron *i* was individually assigned four random numbers, $\lambda _{\mathrm {pre,exc}}$, $\lambda _{\mathrm {post,exc}}$, $\lambda _{\mathrm {pre,inh}}$, and $\lambda _{\mathrm {post,inh}}$, each drawn independently from the interval $[0,\delta ]$, where $\delta =0.2$ is the mean connection density. In a second step, every ordered neuron pair *i*, *j* was individually assigned two random numbers, $\xi$ and $\eta$, drawn independently from [0, 1]. An excitatory projection $j\rightarrow i$ was established, if neuron *j* was excitatory and $\xi <\lambda _{\mathrm {pre,exc}}$. Similarly, an inhibitory projection $j\rightarrow i$ was established, if *j* was inhibitory and $\xi <\lambda _{\mathrm {pre,inh}}$. Projections $i \rightarrow j$ were established if *j* was excitatory and $\eta <\lambda _{\mathrm {post,exc}}$, or if *j* was inhibitory and $\eta <\lambda _{\mathrm {post,inh}}$. This procedure resulted in a random graph with mean connection density of $20~\%$. Heterogeneity arises because each neuron exhibits an individual connection density, with independent out-degree and in-degree.

Established projections were assigned a synaptic weight $w_{ ij }$, each chosen randomly and independently from a (clipped) Gaussian distribution with mean $\omega$ and standard deviation $\omega /2$ (clipping ensured $w_{ ij }>0$). Not established projections were assigned $w_{ ij }=0$. Mean values were chosen such as to obtain spontaneous activity with pronounced synchronization events (‘network spikes’, see below) at rates of $O(10^0\,\mathrm {Hz})$. Specifically, we chose $\omega _\mathrm {ee,0}=1150\,\mathrm {pS}$, $\omega _\mathrm {ei,0}=8500\,\mathrm {pS}$, $\omega _\mathrm {ie,0}=5\,\mathrm {pS}$, and $\omega _\mathrm {ii,0}=200\,\mathrm {pS}$ for homogeneous random networks; $\omega _\mathrm {ee,0}=1450\,\mathrm {pS}$, $\omega _\mathrm {ei,0}=9500\,\mathrm {pS}$, $\omega _\mathrm {ie,0}=5\,\mathrm {pS}$, and $\omega _\mathrm {ii,0}=200\,\mathrm {pS}$ for scale-free networks; and $\omega _\mathrm {ee,0}=1000\,\mathrm {pS}$, $\omega _\mathrm {ei,0}=8500\,\mathrm {pS}$, $\omega _\mathrm {ie,0}=5\,\mathrm {pS}$, and $\omega _\mathrm {ii,0}=200\,\mathrm {pS}$ for heterogeneous random networks.

Almost all realizations of random connectivity resulted in spontaneous network activity including large synchronization events. This was the case for $\approx 90~\%$ of the homogeneous random networks, $\approx 80~\%$ of the scale-free networks, and $\approx 100~\%$ of the heterogeneous random networks. In the remaining realizations, spontaneous activity failed to ignite network spikes (in an all-or-none fashion). The activity of our three networks was asynchronous irregular as shown by the high frequency limit of the respective power spectral densities of the unfiltered population activity (cf. Fig 1c) (Spiridon and Gerstner 1999).

### Power spectra and cross-correlation

For the computation of power spectra and cross-correlations, we divided a long simulation (${\mathscr {O}}(10^3) \,\, \mathrm {s}$, resolution $0.1 \,\, \mathrm {ms}$, with NS removed) into bins of length $T = 20 \,\, s$, thus creating an ensemble of $\approx 50$ time traces. For computation of power spectra of individual neurons, we computed the Fourier transform

12 $$\begin{aligned} {\tilde{\rho }}_{i}(\omega ) = \int \! \mathrm {d}t \rho _i(t) \exp {(\mathrm {i} \omega t)} \end{aligned}$$

of the spike trains of each neuron *i*, integrated over single bins. The power spectrum $S_i(f)$ of the activity of the *i*th excitatory neuron was then determined as

13 $$\begin{aligned} S_i(f) = \frac{\left\langle | {\tilde{\rho }}_i(2 \pi f) |^2 \right\rangle }{T}, \end{aligned}$$

where the average is taken over the ensemble of bins. This was averaged over excitatory neurons to yield Fig. 1c.

For the computation of cross-correlations, we determined the normalized ‘scalar product’

14 $$\begin{aligned} C_{ij}(\tau ) = \frac{\left\langle \int \! \mathrm {d} t \rho _i(t) \rho _j(t+\tau ) \right\rangle }{\sqrt{\left\langle \int \! \mathrm {d} t \rho _i^2(t) \right\rangle } \sqrt{\left\langle \int \! \mathrm {d} t \rho _j^2(t) \right\rangle }} \end{aligned}$$

for each pair of distinct neurons *i* and *j* which discharge at least once between NS. The integral is again over single bins, and the average is over the ensemble of all bins. Spike trains were regularized by square-shaped kernels with a width of $0.5 \,\, \mathrm {ms}$. Then we considered the ensemble of cross-correlations $C_{ij}(0)$ at lag $\tau = 0$ for each pair of distinct excitatory neurons which both discharge between NS. For this ensemble, the mean and standard deviation were stated.

### Histograms and densities

Histograms and densities were computed as follows. In Figs. 1b and 2a, rectangular bins of, respectively, $50\,\mathrm {ms}$ and 2 (relative activity) were used. In all other cases, densities were estimated with Gaussian kernels. Kernel width and sampling resolution for population activity (spike density) were $3\,\mathrm {ms}$ and $0.1\,\mathrm {ms}$, respectively. For latency distributions, the corresponding values were $0.8\,\mathrm {ms}$ and $0.1\,\mathrm {ms}$, with approximately 400 samples per kernel. For voltage distributions, they were $0.1\,\mathrm {mV}$ and $10\,\mathrm {\mu V}$, with 20, 000 samples. For the fraction of recovered resources *R*, kernel width and voltage distribution were 0.05 and 0.01, with $O(10^4)$ samples.

Recovered resources $R_i(t)$ of neuron *i* were averaged over synapses $i\rightarrow j$ to $N_i$ post-synaptic neurons *j*

15 $$\begin{aligned} R_i(t)=\frac{\sum _j R_{ji}(t)}{N_i}, \end{aligned}$$

where $R_{ji}(t)$ is recovered resources of synapse $i\rightarrow j$ at time *t*. Densities $p_i(R)$ were established over all time points *excluding* NS (i.e. time points more than $35\,\mathrm {ms}$ before or after a NS).

Population activity is understood to be activity of the excitatory subpopulation and is sometimes given as *absolute* activity (in $\mathrm {Hz}$) and sometimes as *relative* activity (i.e. in units of the average activity level).

### Network spikes, peak activities, and synchrony thresholds

Network spikes (NSs) are large bursts of excitatory activity separated by long periods of low activity. We defined NS with respect to a high threshold (half the maximal activity): $\theta _\mathrm {high} = 0.5 \cdot \mathrm {max}[A(t)]$, where *A*(*t*) is excitatory activity in $\mathrm {Hz}$. Beginning and end $[t_i,t_f]$ of a NS were defined as successive crossings of $\theta _\mathrm {high}$ by *A*(*t*) from below and from above. For each NS, we determined duration $t_{\mathrm {NS}}=t_f-t_i$ and peak activity $A_{\mathrm {max}}$.

Note that this definition captures only large bursts of activity. Smaller fluctuations were detected with a lower threshold $\theta _\mathrm {low} = 1.1 \cdot \mathrm {mean}(A)$. The distribution of peak activities $A_{\mathrm {max}}$ could be monomodal or bimodal. Bimodal distributions indicated ‘all-or-none’ synchronization events, consistent with a super-critical regime (Gigante et al. 2015). Typically, probability density was divided between low values ($< 10$ times mean activity) and high values ($>40$ times mean activity), with zero density in between.

The largest ‘low’ values constitute a lower bound for the ‘threshold’ of NS initiation, as any intermediate values must have resulted in ‘runaway’ amplification to a ‘high’ value. Specifically, we determined this ‘threshold’ as the largest observed value of the lower concentration of probability mass. In sufficiently long simulations, this empirical value should provide a close lower bound for the ‘true’ threshold.

To characterize activity between NS, we exclude NS by omitting $35\,\mathrm {ms}$ of activity before and after peak activity. This value reflects the shape of NS, which is highly stereotypical (not shown).

### Encoding of external stimulation

To assess the extent to which network activity encodes external stimulation, we perturbed spontaneous network activity in some simulations. External stimulation targeted particular subsets of excitatory neurons (10 or 30 randomly selected neurons) and forced a single spike in each target neuron. Each subset of targets was considered a ‘stimulation site’ and up to 12 non-overlapping sites were used. Subsequent network activity (i.e. $100\,\mathrm {ms}$ of activity, exclusive of the forced spikes) was characterized in terms of four features, following (Kermany et al. 2010). Two features were based on firing rates $a_i(t)$ of neurons *i*: temporal profile of population activity $A(t) = \sum _i a_i(t)$, and spatial profile of population activity $A_i = \int a_i(t) \mathrm{d}t$. Two further features were based on spiking activity of neurons *i* in the interval between stimulation and the subsequent NS: timing of first spikes $t_i$, and rank order of first spikes $o_i$. Rank order was obtained by sorting negative spike latencies with respect to the subsequent NS (for example, the negative latency vector $(-20\,\mathrm {ms},-10\,\mathrm {ms}, -15\,\mathrm {ms}, -17\,\mathrm {ms})$ would yield the rank order vector (1, 4, 3, 2)).

To analyse the information encoded by different activity features, simulations were divided into a training and a test set. Following (Kermany et al. 2010), the training set was used to train a support vector machine (SVM, from Python library LIBSVM, module ‘sklearn’ (Chang and Lin 2011)) to classify the stimulated location on the basis a particular feature. The test set was used to determine classification performance (fraction of correct classifications of the stimulated site), providing a lower bound for the ‘true’ information about stimulation site encoded by a particular activity feature.

### Silencing of neurons

To assess the relative importance of different subsets of excitatory neurons, we wished to render ineffective the members of any particular subset. To do so, we retained the spikes of such neurons but suppressed all post-synaptic effects. A reduced frequency of NS in partially de-afferentiated networks revealed the relative importance of the manipulated subset of neurons.

### Spike-triggered average population activity

To assess the relation between population activity and individual neuron spikes, we computed the ‘spike-triggered deviation’ $\varGamma _i(\tau )$ as follows

16 $$\begin{aligned} \varGamma _i(\tau ) = \frac{\int \left( A(t) - \left\langle A \right\rangle \right) \rho _i(t-\tau ) \mathrm {d} t}{\int \rho _i(t) \mathrm {d}t}, \end{aligned}$$

where *A*(*t*) is time-dependent population activity, $\left\langle A \right\rangle$ is its temporal mean, $\rho _i(t)$ is the spike sequence (Dirac comb) of neuron *i*, and $\tau$ is the latency between activity time *t* and spike time $t-\tau$. The computation was restricted to periods between NS and the normalization term $\int \rho _i(t) \mathrm {dt}$ is the number of spikes fired by neuron *i* between NS. In principle, $\varGamma _i(\tau )$ measures *influence* on ($\tau >0$), as well as sensitivity to ($\tau <0$), population activity of neuron *i*. However, as many neurons spike only shortly before NS, we mostly obtain information about negative latencies, that is, about sensitivity to population activity. For this reason, the spike-triggered deviation in Fig. 4c is restricted to negative latencies. Moreover, it is defined only for (sorted) neuron ID $>260$, as less active neurons never spike between NS.

### Estimation of post-synaptic effects

To estimate the differential effect of neuron *i* on post-synaptic neurons *j* throughout the network, we proceeded as follows. For every synaptic target *j*, we formed the difference $W_ ji \equiv V'_j - V_j$ between the hypothetical star voltage $V'_j(t)$ that would have resulted from a single additional spike of neuron *i* at time $t_\mathrm {sp}$ and the actual the star voltage $V_j(t)$, which may be approximated as

17 $$\begin{aligned} W_ ji (t)&\approx \frac{\tau _\mathrm{I} U w R_\mathrm{m} (E_\mathrm {exc}- \left\langle V_j\right\rangle )}{(\tau _\mathrm{m} - \tau _\mathrm{I}) (1 + U \tau _\mathrm {rec} \nu _i)} \varTheta (t-t_\mathrm {sp})\nonumber \\&\quad \left[ e^{ -(t-t_\mathrm {sp}) / \tau _\mathrm{m} } - e^{ -(t-t_\mathrm {sp})/\tau _\mathrm{I} }\right] , \end{aligned}$$

where $\nu _i$ is the asynchronous firing rate of neuron *i* (between NS), $\left\langle V_j \right\rangle$ is the expected star voltage of neuron *j*, and $\tau _\mathrm{I}$, *U*, *w*, and $\tau _\mathrm {rec}$ are parameters of the synapse in question. Note that we neglect conduction delays and assume the driving force to be constant. The expression for $W_ ji (t)$ peaks at time

18 $$\begin{aligned} t_\mathrm {max} = \frac{\tau _\mathrm{m} \tau _\mathrm{I} }{(\tau _\mathrm{m} - \tau _\mathrm{I})} \log { \left( \frac{\tau _\mathrm{m}}{\tau _\mathrm{I}} \right) } , \end{aligned}$$

so that the post-synaptic potential in neuron *j* that is triggered by the additional spike in neuron *i* at time $t_\mathrm {sp}$ is $W_ ji (t_\mathrm {max})$. The cumulative post-synaptic effect of all spikes in neuron *i* is given by the stationary limit

19 $$\begin{aligned} \left\langle W_ ji \right\rangle _\mathrm {ss} = \frac{R_\mathrm{m} w (E_\mathrm {exc}-\left\langle V_j\right\rangle ) \tau _\mathrm{I} U \nu _i}{1+U \tau _\mathrm {rec} \nu _i}, \end{aligned}$$

which is approximately equal to $\tau _\mathrm{m} \cdot \nu _i \cdot W_ ji (t_\mathrm {max})$.

In Fig 5b, c, the differential effects of neuron *i* are averaged over all $N_i$ post-synaptic neurons *j*

20 $$\begin{aligned} \mathrm {PSP}_i \equiv \frac{1}{N_i}~\sum _j W_{ji}(t_\mathrm {max}), \quad \quad \left\langle \mathrm {PSP}_i \right\rangle _\mathrm {ss} \equiv \frac{1}{N_i}~\sum _j \left\langle W_{ji} \right\rangle _\mathrm {ss}. \end{aligned}$$

In Fig. 5d, e, the products $\mathrm {PSP}_i \cdot N_i$ and $\left\langle \mathrm {PSP}_i \right\rangle _\mathrm {ss} N_i$ are plotted (summed effect).

### Modification of Levenshtein edit distance

To quantify dissimilarity in the rank order or ‘first spikes’ observed in different contexts, we modified the Levenshtein edit distance (Levenshtein 1966) used in previous studies (Shahaf et al. 2008). The Levenshtein metric is useful for strings with same and/or different ‘letters’; in the present situation, all rank order strings contain the same ‘letters’ (because all neurons fire at least one spike and rare missing spikes can be ‘filled in’ at the highest rank). Now consider two strings $s_1s_2 \dots s_n$ and $s_{\pi (1)} s_{\pi (2)} \dots s_{\pi (n)}$, where $\pi$ is an appropriately chosen permutation. Then the number of inversions *L*, which is the number pairs (*i*, *j*) such that $i<j$ but $\pi (i)>\pi (j)$, ranges from 0 (if strings are identical) to $L=\frac{n(n-1)}{2}$ (if strings are inverted). Accordingly, we adopted

21 $$\begin{aligned} L_n = \left( 1 - \frac{2L}{n(n-1)} \right) ~100 \% \end{aligned}$$

as normalized measure of similarity.

### Determination of the fraction of pioneer neurons

We determined the number of pioneers at different points in the excitation–inhibition landscape as follows (Fig. 11a). Pioneers should have small $CV(\tau )$ and large $CV(V^\star )$. Consequently, we adopted an operational criterion and counted all neurons as pioneers which have $CV(\tau ) < 0.64$ and $|CV(V^\star )| > 0.64$. The choice of these two values approximately detected the neurons with sorted ID between 260 and 320 as the pioneers in the heterogeneous case (at relative weights $r_\mathrm{E}=r_\mathrm{I}=1$).

### Toy theory for latency statistics

The comparison of latency statistics with the ones expected from *identical* time course of population activity (Fig. 13c, d) proceeded as follows. The idea was to obtain a simple and qualitative prediction for latency distributions under the assumption that the activity shape of NS and hence the ‘activation function’ of individual neurons *are identical*, so that a first-order prediction for the characteristic deviation of latencies is obtained.

To simplify as much as possible, we employed a ‘toy theory’ in which the membrane voltage of all neurons undergoes an identical, time-dependent translation *u*(*t*) along the voltage axis. Specifically, the distribution of $\star$ voltage changes over time

22 $$\begin{aligned} p(t,V) = \frac{1}{\sqrt{2 \pi \sigma ^2}} \exp {\left( - \frac{\left[ V - \left\langle V \right\rangle - u(t)\right] ^2}{2 \sigma ^2} \right) }, \end{aligned}$$

as prescribed by

23 $$\begin{aligned} u(t) = \alpha \exp {\left( - \frac{t^2}{2 \sigma _\mathrm{NS}^2} \right) }. \end{aligned}$$

Here, $\sigma$ is the standard deviation of the membrane star voltage, $\left\langle V \right\rangle$ is the average level of the same quantity between NS, and $\alpha = 0.56 \,\, \mathrm {mV}$ and $\sigma _\mathrm{NS}= 23 \,\, \mathrm {ms}$ determine the shape of the activation function *u*(*t*). The values were identical for all topologies, but were chosen to approximate the comparatively slow time course of NS in heterogeneous networks.

Next, we approximate the firing statistics relative to NS by the probability flux through threshold, if it is positive, and by zero otherwise:

24 $$\begin{aligned} p(\tau ) = \left[ \frac{\mathrm {d}}{\mathrm {d} \tau } \int _{V_{\vartheta }}^\infty \!\! p(\tau ,V) \mathrm {d} V \right] _+, \end{aligned}$$

where $[\cdot ]_+$ denotes half-wave rectification. The resulting simple theory reads

25 $$\begin{aligned} p(\tau ) = N [{\dot{\mu }}(\tau )]_+ \exp {\left( - \frac{\left[ V_{\vartheta }- \left\langle V \right\rangle - u(\tau )\right] ^2}{2 \sigma ^2} \right) }, \end{aligned}$$

where *N* is a normalization factor, and was used in order to obtain simple predictions for the statistics of the latency of individual neurons.

## Electronic supplementary material

Below is the link to the electronic supplementary material.

Supplementary material 1 (pdf 118 KB)

## References

[CR1] Ansmann G, Karnatak R, Lehnertz K, Feudel U (2013). Extreme events in excitable systems and mechanisms of their generation. Phys Rev E.

[CR2] Bak P, Tang C, Wiesenfeld K (1988). Self-organized criticality. Phys Rev A.

[CR3] Barabási AL, Albert R (1999). Emergence of scaling in random networks. Science.

[CR4] Bauermeister C, Keren H, Braun J (2015) Coherent coupling of in vitro neuronal slices onto in silico networks. In: 11th Bernstein conference, Heidelberg, Germany

[CR5] Beggs JM, Plenz D (2003). Neuronal avalanches in neocortical circuits. J Neurosci.

[CR6] Brunel N (2000). Dynamics of sparsely connected networks of excitatory and inhibitory spiking neurons. J Comput Neurosci.

[CR7] Chang CC, Lin CJ (2011). LIBSVM: a library for support vector machines. ACM Trans Intell Syst Technol (TIST).

[CR8] Contreras EJB, Schjetnan AGP, Muhammad A, Barthó P, McNaughton BL, Kolb B, Gruber AJ, Luczak A (2013). Formation and reverberation of sequential neural activity patterns evoked by sensory stimulation are enhanced during cortical desynchronization. Neuron.

[CR9] Decharms RC, Zador A (2000). Neural representation and the cortical code. Annu Rev Neurosci.

[CR10] Effenberger F, Jost J, Levina A (2015). Self-organization in balanced state networks by STDP and homeostatic plasticity. PLoS Comput Biol.

[CR11] Eytan D, Marom S (2006). Dynamics and effective topology underlying synchronization in networks of cortical neurons. J Neurosci.

[CR12] Feng J (2003). Computational neuroscience: a comprehensive approach.

[CR13] Gerstner W, Kistler WM, Naud R, Paninski L (2014). Neuronal dynamics: from single neurons to networks and models of cognition.

[CR14] Gigante G, Deco G, Del Giudice P (2014) Spontaneous and evoked population bursts: history-dependent response, differential role of neuronal adaptation, synaptic short-term depression, and time scales inference. In: 9th FENS forum of neuroscience, Milan, Italy

[CR15] Gigante G, Deco G, Marom S, Del Giudice P (2015). Network events on multiple space and time scales in cultured neural networks and in a stochastic rate model. PLoS Comput Biol.

[CR16] Gritsun T, Stegenga J, Le Feber J, Rutten W (2008) Explaining burst profiles using models with realistic parameters and plastic synapses. In: MEA meeting 2008, p 26

[CR17] Gritsun TA, Le Feber J, Stegenga J, Rutten WL (2010). Network bursts in cortical cultures are best simulated using pacemaker neurons and adaptive synapses. Biol Cybern.

[CR18] Gritsun T, le Feber J, Stegenga J, Rutten WL (2011). Experimental analysis and computational modeling of interburst intervals in spontaneous activity of cortical neuronal culture. Biol Cybern.

[CR19] Harrison PM, Badel L, Wall MJ, Richardson MJ (2015). Experimentally verified parameter sets for modelling heterogeneous neocortical pyramidal-cell populations. PLoS Comput Biol.

[CR20] Jaeger H, Haas H (2004). Harnessing nonlinearity: predicting chaotic systems and saving energy in wireless communication. Science.

[CR21] Jensen HJ (1998). Self-organized criticality: emergent complex behavior in physical and biological systems.

[CR22] Kermany E, Gal A, Lyakhov V, Meir R, Marom S, Eytan D (2010). Tradeoffs and constraints on neural representation in networks of cortical neurons. J Neurosci.

[CR23] Koch C (1999). Biophysics of computation: processing in single neurons.

[CR24] Landau ID, Egger R, Dercksen VJ, Oberlaender M, Sompolinsky H (2016). The impact of structural heterogeneity on excitation-inhibition balance in cortical networks. Neuron.

[CR25] Levenshtein VI (1966). Binary codes capable of correcting deletions, insertions, and reversals. Sov Phys Dokl.

[CR26] Levy O, Ziv NE, Marom S (2012). Enhancement of neural representation capacity by modular architecture in networks of cortical neurons. Eur J Neurosci.

[CR27] Loebel A, Tsodyks M (2002). Computation by ensemble synchronization in recurrent networks with synaptic depression. J Comput Neurosci.

[CR28] Luccioli S, Ben-Jacob E, Barzilai A, Bonifazi P, Torcini A (2014). Clique of functional hubs orchestrates population bursts in developmentally regulated neural networks. PLoS Comput Biol.

[CR29] Luczak A, Barthó P (2012). Consistent sequential activity across diverse forms of up states under ketamine anesthesia. Eur J Neurosci.

[CR30] Luczak A, MacLean JN (2012). Default activity patterns at the neocortical microcircuit level. Front Integr Neurosci.

[CR31] Luczak A, Barthó P, Marguet SL, Buzsáki G, Harris KD (2007). Sequential structure of neocortical spontaneous activity in vivo. Proc Natl Acad Sci.

[CR32] Lukosevicius M, Jaeger H (2009). Reservoir computing approaches to recurrent neural network training. Comput Sci Rev.

[CR33] Maass W, Natschläger T, Markram H (2002). Real-time computing without stable states: a new framework for neural computation based on perturbations. Neural Comput.

[CR34] Marom S, Shahaf G (2002). Development, learning and memory in large random networks of cortical neurons: lessons beyond anatomy. Q Rev Biophys.

[CR35] Masquelier T, Deco G (2013). Network bursting dynamics in excitatory cortical neuron cultures results from the combination of different adaptive mechanism. PLoS ONE.

[CR36] Matsumoto K, Ishikawa T, Matsuki N, Ikegaya Y (2013). Multineuronal spike sequences repeat with millisecond precision. Front Neural Circuits.

[CR37] Mattia M, Del Giudice P (2002). Population dynamics of interacting spiking neurons. Phys Rev E.

[CR38] Morin FO, Takamura Y, Tamiya E (2005). Investigating neuronal activity with planar microelectrode arrays: achievements and new perspectives. J Biosci Bioeng.

[CR39] Pasquale V, Massobrio P, Bologna L, Chiappalone M, Martinoia S (2008). Self-organization and neuronal avalanches in networks of dissociated cortical neurons. Neuroscience.

[CR40] Pena RFO, Vellmer S, Bernardi D, Roque AC, Lindner B (2018). Self-consistent scheme for spike-train power spectra in heterogeneous sparse networks. Front Comput Neurosci.

[CR41] Persi E, Horn D, Segev R, Ben-Jacob E, Volman V (2004). Neural modeling of synchronized bursting events. Neurocomputing.

[CR42] Persi E, Horn D, Volman V, Segev R, Ben-Jacob E (2004). Modeling of synchronized bursting events: the importance of inhomogeneity. Neural Comput.

[CR43] Peyrache A, Benchenane K, Khamassi M, Wiener SI, Battaglia FP (2010). Sequential reinstatement of neocortical activity during slow oscillations depends on cells’ global activity. Front Syst Neurosci.

[CR44] Poil SS, Hardstone R, Mansvelder HD, Linkenkaer-Hansen K (2012). Critical-state dynamics of avalanches and oscillations jointly emerge from balanced excitation/inhibition in neuronal networks. J Neurosci.

[CR45] Ponulak F, Kasinski A (2011). Introduction to spiking neural networks: information processing, learning and applications. Acta Neurobiol Exp.

[CR46] Priesemann V, Wibral M, Valderrama M, Pröpper R, Le Van Quyen M, Geisel T, Triesch J, Nikolic D, Munk MH (2014). Spike avalanches in vivo suggest a driven, slightly subcritical brain state. Front Syst Neurosci.

[CR47] Rajan K, Harvey CD, Tank DW (2016). Recurrent network models of sequence generation and memory. Neuron.

[CR48] Rieke F (2008). Spikes: exploring the neural code.

[CR49] Rolls E (2008). Memory, attention and decision-making.

[CR50] Rolls E, Deco G (2010). The noisy brain: stochastic dynamics as a principle of brain function.

[CR51] Rolston JD, Wagenaar DA, Potter SM (2007). Precisely timed spatiotemporal patterns of neural activity in dissociated cortical cultures. Neuroscience.

[CR52] Shahaf G, Eytan D, Gal A, Kermany E, Lyakhov V, Zrenner C, Marom S (2008). Order-based representation in random networks of cortical neurons. PLoS Comput Biol.

[CR53] Shepherd GM (2003). The synaptic organization of the brain.

[CR54] Spiridon M, Gerstner W (1999). Noise spectrum and signal transmission through a population of spiking neurons. Netw Comput Neural Syst.

[CR55] Stark E, Roux L, Eichler R, Buzsáki G (2015). Local generation of multineuronal spike sequences in the hippocampal CA1 region. Proc Natl Acad Sci.

[CR56] Thorpe S, Delorme A, Van Rullen R (2001). Spike-based strategies for rapid processing. Neural Netw.

[CR57] Tsodyks M, Pawelzik K, Markram H (1998). Neural networks with dynamic synapses. Neural Comput.

[CR58] Tsodyks M, Uziel A, Markram H (2000). Synchrony generation in recurrent networks with frequency-dependent synapses. J Neurosci.

[CR59] Tuckwell HC (2005). Introduction to theoretical neurobiology.

[CR60] Vladimirski BB, Tabak J, O’Donovan MJ, Rinzel J (2008). Episodic activity in a heterogeneous excitatory network, from spiking neurons to mean field. J Comput Neurosci.

[CR61] Wiedemann UA, Lüthi A (2003). Timing of network synchronization by refractory mechanisms. J Neurophysiol.

[CR62] Wills P, Meyer FG (2019) Metrics for graph comparison: a practitioner’s guide. arXiv preprint arXiv:1904.07414 10.1371/journal.pone.0228728 PMC7015405 32050004

[CR63] Zbinden C (2011). Leader neurons in leaky integrate and fire neural network simulations. J Comput Neurosci.

