# Supplementary figures and images for: Unstructured network topology begets order-based representation by privileged neurons
Source: Biol Cybern. 2020 Feb 27;114(1):113–35. doi: 10.1007/s00422-020-00819-9 (PMC7062672; doi:10.1007/s00422-020-00819-9)

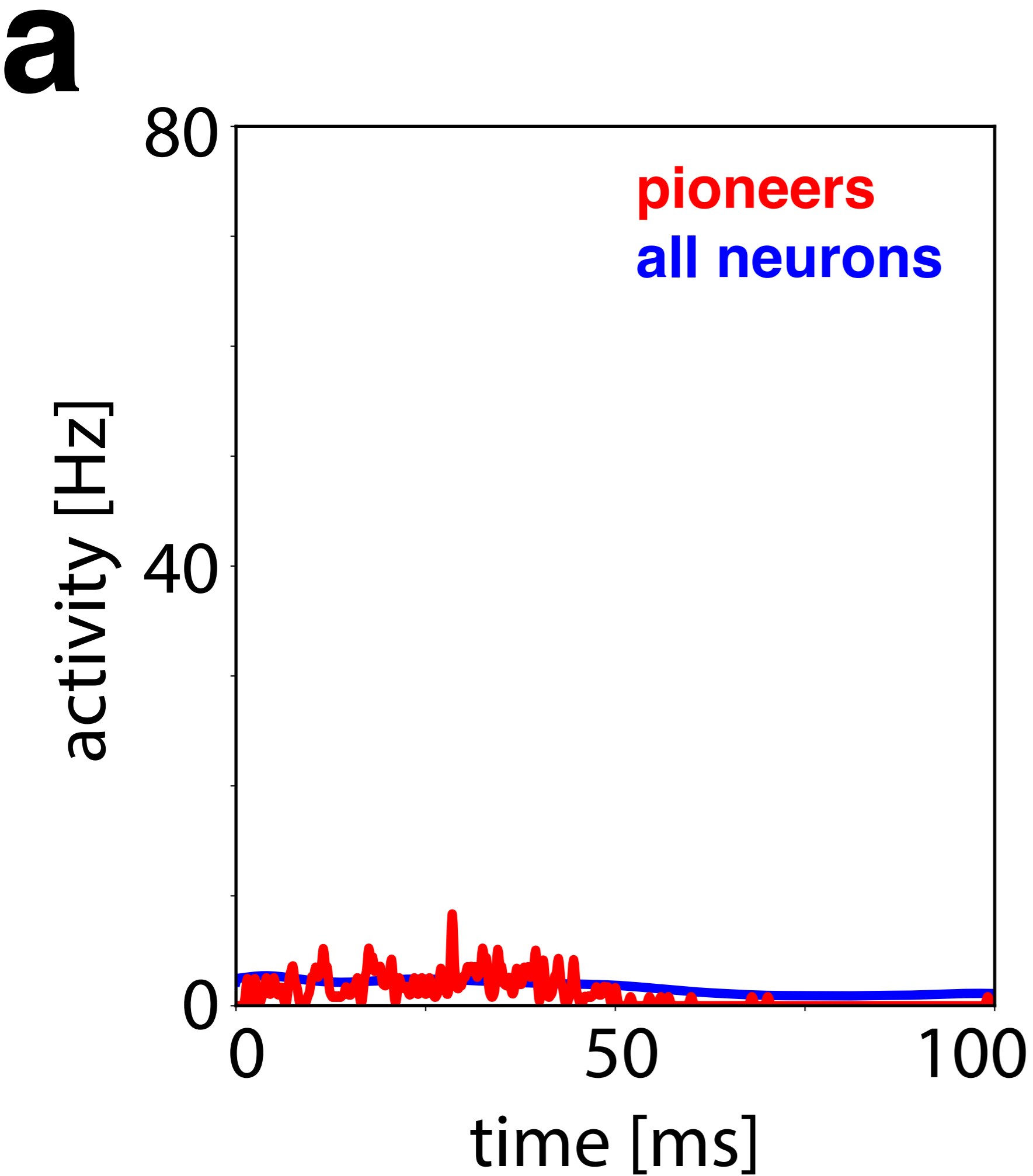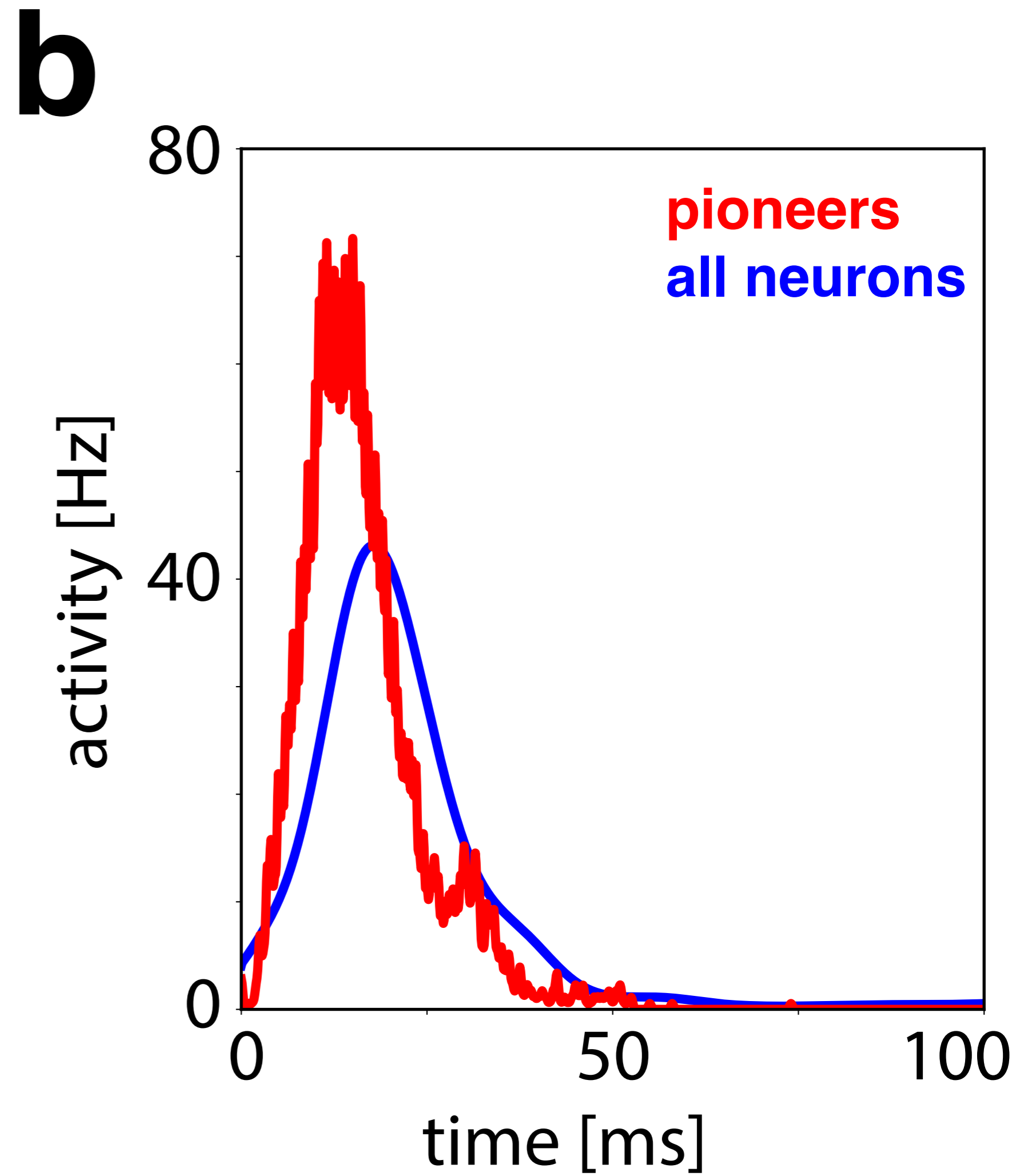

Supplement: Supplementary file 1 — Supplementary material 1 (pdf 118 KB) [file 422_2020_819_MOESM1_ESM.pdf]
